# FAK mediates LPS-induced inflammatory lung injury through interacting TAK1 and activating TAK1-NFκB pathway

**DOI:** 10.1038/s41419-022-05046-7

**Published:** 2022-07-08

**Authors:** Xi Chen, Ying Zhao, Xu Wang, Yimin Lin, Weixin Zhao, Di Wu, Jingye Pan, Wu Luo, Yi Wang, Guang Liang

**Affiliations:** 1 Chemical Biology Research Center, School of Pharmaceutical Science, Wenzhou Medical University, Wenzhou, Zhejiang 325035 China; 2 Department of Pharmacology, Medical College, Taizhou University, Taizhou, Jiaojiang, 318000 Zhejiang China; 3 Department of Critical Care Medicine and Medical Research Center, the First Affiliated Hospital of Wenzhou Medical University, Wenzhou, Zhejiang 325000 China; 4 School of Pharmaceutical Sciences, Hangzhou Medical College, Hangzhou, Zhejiang 311399 China; 5 Wenzhou Institute, University of Chinese Academy of Sciences, Wenzhou, Zhejiang 325001 China

**Keywords:** Bacterial infection, Phosphoinositol signalling

## Abstract

Acute lung injury (ALI), characterized by inflammatory damage, is a major clinical challenge. Developing specific treatment options for ALI requires the identification of novel targetable signaling pathways. Recent studies reported that endotoxin lipopolysaccharide (LPS) induced a TLR4-dependent activation of focal adhesion kinase (FAK) in colorectal adenocarcinoma cells, suggesting that FAK may be involved in LPS-induced inflammatory responses. Here, we investigated the involvement and mechanism of FAK in mediating LPS-induced inflammation and ALI. We show that LPS phosphorylates FAK in macrophages. Either FAK inhibitor, site-directly mutation, or siRNA knockdown of FAK significantly suppresses LPS-induced inflammatory cytokine production in macrophages. FAK inhibition also blocked LPS-induced activation of MAPKs and NFκB. Mechanistically, we demonstrate that activated FAK directly interacts with transforming growth factor-β-activated kinase-1 (TAK1), an upstream kinase of MAPKs and NFκB, and then phosphorylates TAK1 at Ser412. In a mouse model of LPS-induced ALI, pharmacological inhibition of FAK suppressed FAK/TAK activation and inflammatory response in lung tissues. These activities resulted in the preservation of lung tissues in LPS-challenged mice and increased survival during LPS-induced septic shock. Collectively, our results illustrate a novel FAK-TAK1-NFκB signaling axis in LPS-induced inflammation and ALI, and support FAK as a potential target for the treatment of ALI.

## Introduction

Acute lung injury (ALI) is associated with high rates of morbidity and mortality [1–3]. ALI is a continuum of pathological changes and leads to acute respiratory distress syndrome (ARDS). A major cause of ALI and ARDS is sepsis [4, 5]. During the early injury phase, epithelial cell apoptosis and destruction of the basement membranes are seen [6]. This loss of epithelial integrity is coupled with increased capillary permeability leading to an influx of plasma proteins into the alveolar space. There is subsequent leukocyte infiltration [7, 8] and exacerbation of inflammatory cytokines [9] that perpetuate the injury. Major proinflammatory cytokines in this context include tumor necrosis factor alpha (TNF-α), interleukins-1β (IL-1β), and IL-6 [10–12]. Efforts have been made at combating the underlying inflammatory responses in ALI/ARDS to limit injury. Unfortunately, however, there are no specific/adequate pharmacological therapies.

In preclinical models, ALI is produced through the administration of bacterial endotoxin [13]. Lipopolysaccharide (LPS), the main element of Gram-negative bacteria, is now recognized to play a role in ALI and sepsis, and considered an important risk factor [14]. In vivo administration of LPS causes systemic inflammatory responses with ARDS and septic shock. Thus, LPS is able to mimic morphological and functional changes observed in clinical situations. LPS binds to toll-like receptor-4 (TLR4) through accessory proteins including LPS-binding protein, CD14, and myeloid differentiation protein 2 [15–17]. TLR4 then recruits the adaptor molecule myeloid differentiation factor 88 to trigger a pro-inflammatory cascade, including the activation of downstream mitogen-activated protein kinases (MAPKs) and nuclear factor-κB (NFκB), culminating in the elaboration of inflammatory cytokines [18].

Focal adhesion kinase (FAK) is a protein tyrosine kinase that has been found to regulate cellular adhesion, motility, proliferation, and survival in various types of cells. Since FAK is frequently activated and/or overexpressed in advanced cancers and promotes cancer progression and metastasis, it became a potential therapeutic target in cancer. Interestingly, recent studies have identified a novel role of FAK in the TLR4 signaling axis [19] during LPS-induced intestinal permeability alterations. Specifically, researchers showed that LPS caused a TLR4-dependent activation of FAK in human colorectal adenocarcinoma cells, a model of colonic epithelial cells. This FAK activation increased epithelial permeability both in vitro and in mouse models of LPS challenge. TLR4 has also been shown to phosphorylate and activate FAK in necrotizing enterocolitis mouse models [20]. FAK has been also reported to activate NFκB in endothelial cells [3]. These studies suggest that LPS may utilize FAK to induce NFκB and mount an inflammatory response in lung tissues during ALI.

The purpose of the present study was to determine whether LPS regulates FAK in ALI and to discover downstream mechanisms. To achieve this, we utilized one of the predominant infiltrating cells during ALI, macrophages [7, 8]. We challenged the cells in culture and examined signaling pathways that were activated. To confirm the results, we performed intratracheal instillation of LPS in mice and examined the lung tissues. Results of our study reveal that LPS activates FAK in macrophages. Activated FAK then associates with and activates transforming growth factor-β-activated kinase-1 (TAK1), an upstream kinase of NFκB. This signaling axis mediated inflammatory cytokine production and lung injury.

## Materials and methods

### Reagents

Small-molecule FAK inhibitor PND-1186 and TAK1 inhibitor Takinib were purchased from Selleck Chemicals (Houston, TX). LPS was purchased from Sigma-Aldrich (St. Louis, MO, USA). Antibodies against extracellular signal-regulated kinase (ERK), phosphorylated (p)-ERK, total p38, p-p38, c-Jun N-terminal kinase (JNK), p-JNK, p-FAK, total FAK, p-TAK1, total TAK1, inhibitor of κBα (IκBα), p65 subunit of NFκB, p-p65, IκB kinase β (IKKβ), and p-IKKα/β were purchased from Cell Signaling (Danvers, MA, USA). Antibodies against GAPDH and F4/80 were obtained from Santa Cruz Biotechnology (Santa Cruz, CA, USA). Lamin B antibody was purchased from Abcam (Abcam, USA). Secondary antibodies were obtained from Santa Cruz Biotechnology.

### Macrophage culture

RAW 264.7 and THP-1 cell lines were purchased from ATCC (Manassas, VA, USA). RAW 264.7 cells were cultured in DMEM (Gibco, Eggenstein, Germany) containing 5.5 mM of D-glucose; THP-1 cells were cultured in RPMI-1640 and supplemented with 10% fetal bovine serum (FBS; Gibco), 100 U/mL penicillin (Gibco) and 100 mg/mL streptomycin (Gibco). Primary mouse peritoneal macrophages (MPMs) were prepared and cultured from C57BL/6 mice as previously described [21]. Cells were treated as indicated in respective experimental studies.

### Gene silencing and overexpression

Gene knockdowns in RAW 264.7 cells were performed by transfecting cells with siRNA. Cells were plated at a density of 5 × 10^6^ per well in six-well plates and allowed to attach for 20 h. Cells were transfected with 100 nM siRNA against FAK using lipofectamine 2000 (Invitrogen, Carlsbad, CA, USA). After 24 h, cells were treated as indicated. To express FAK in macrophages, a recombinant plasmid vector coding FAK cDNA was obtained from Sino Biological (Cat No.: MG50470-NF). To inactivate FAK by site-directed mutation, the FAK Tyr397 to Phe397 variant (Y397F) obtained from TSINGKE Biological (Nangjing, China) was used. These plasmids were transfected into RAW 264.7 cells using Lipofectamine 3000.

To overexpress FAK, FAK mutation, and TAK1 in tool cells, three plasmids respectively encoding Flag-labeled wide-type FAK (FAK-WT-Flag), Flag-labeled FAK Y397F mutation (FAK-Y397F-Flag), and Myc-labeled TAK1 (TAK1-Myc) were designed and obtained from OriGene Technologies (Beijing, China). These three plasmids were transfected into 3T3 cells using lipofectamine 3000.

### NFκB reporter assays

RAW 264.7 cells stably expressing NFκB-RE-EGFP reporter were prepared by lentivirus infection. Lentivirus containing the response element of NFκB was first generated by co-transfecting HEK293T cells with p-LV-NFκB-RE-EGFP (Inovogen) and packaging plasmids (psPAX2 and pMD2.G) using PEI (polysciences). The supernatant was collected 48 h later and filtered using a 0.45 μm filter. Then, RAW 264.7 cells were incubated with supernatant and 8 mg/mL polybrene (polysciences) for 12 h. Cells were selected with 2 mg/mL puromycin (Invitrogen, San Diego) for 10 days. Following various treatments, NFκB activity was detected by Accuri C6 plus flow cytometry (BD Biosciences, CA, USA).

### Immunofluorescence cell staining

RAW 364.7 and MPM cells were treated as indicated. Following treatments, cells were fixed in cold methanol. Cells were then incubated with primary antibodies for 1 h at room temperature. Secondary Alexa488 conjugated antibodies were used for detection. Cell slides were counterstained with DAPI. Images were captured using Leica Confocal Laser Scanning Microscope (Leica, Germany).

### Cytokine levels

Levels of TNF-α and IL-6 in cell culture medium, bronchoalveolar lavage fluid (BALF), and serum from C57BL/6 mice were determined with an ELISA kit (Bioscience, San Diego, CA, USA). MPMs, RAW 264.7, and THP-1 cells were seeded in six-well plates at a density of 400,000 cells per well. Following treatments, media were collected to measure the amount of TNF-α and IL-6. Levels of cytokines were normalized to total proteins in the same samples.

### Real-time quantitative PCR

Total RNA was extracted from cells and tissues using TRIZOL (Thermo Fisher). Reverse transcription and quantitative PCR was carried out using a two-step M-MLV Platinum SYBR Green qPCR SuperMix-UDG kit (Thermo Fisher) and Eppendorf Mastercycler ep realplex detection system (Eppendorf, Hamburg, Germany). Primers for TNF-α, IL-6, IL-1β, COX-2, and β-actin were obtained from Thermo Fisher. Primer sequences are shown in Supplementary Table 1. The relative amount of each gene was normalized to β-actin.

### Cell-free kinase assay

Recombinant human TAK1 protein (rhTAK1) and recombinant human FAK kinase domain (rhFAK) were expressed and obtained from Zeye Biotechnology (Shanghai, China). rhTAK1 (10 ng/μL) was incubated with 4 ng/μL of rhFAK in kinase assay buffer containing 50 mM Hepes (pH 7.5), 1 mM EGTA, 0.01 mM BSA, 2 mM MnCl_2_, 10 mM MgCl_2_, 2 mM DTT, 0.01% Tween 20, with or without 100 μM ATP (Cell Signaling, #9804). The incubation was performed for 30 min at 37 °C. The samples were boiled at 100 °C for 10 min and separated by sodium dodecyl sulfate polyacrylamide gel electrophoresis (SDS-PAGE), and then transferred onto a polyvinylidene fluoride membrane. Signals for p-rhTAK1, rhTAK1, and rhFAK were detected using the respective antibodies.

### Western blotting

Cell lysates were subjected to 8–12% SDS-PAGE and transferred onto a PVDF membrane (Bio-Rad Laboratories). Membranes were blocked in 5% milk in tris-buffered saline, containing 0.05% Tween 20 for 1.5 h at room temperature. Membranes were then incubated with different primary antibodies overnight at 4 °C. The membranes were washed in TBS-T and incubated with secondary horseradish peroxidase-conjugated antibodies (Santa Cruz, CA, USA; 1:5000) for 2 h at room temperature. Blots were then visualized using enhanced chemiluminescence reagents (Bio-Rad Laboratories). The density of the immunoreactive bands was analyzed using Image J software (NIH, Bethesda, MD, USA).

Nuclear proteins, where indicated, were prepared using a cytoplasmic and nuclear protein extraction kit (KeyGEN, Nanjing, China). Levels of nuclear p65 subunit were probed using Western blotting.

For immunoprecipitation studies, TAK1 was co-precipitated with FAK from macrophages to detect the association of TAK1 with FAK. Cell extracts were incubated with anti-FAK antibody for 1 h and then precipitated with protein G-Sepharose beads at 4 °C overnight. TAK1 levels were detected by immunoblotting.

### LPS-induced inflammatory lung injury and sepsis in mice

Six-week-old male C57BL/6 (totally *n* = 58) were obtained from the Wenzhou Medical University Animal Center. Protocols involving the use of the animals were approved by the Wenzhou Medical University Animal Policy and Welfare Committee (Approved number: wydw2019-0013). All animals were housed in a pathogen-free room under 22 ± 2 °C, 50–60% humidity, 12:12 h light-dark cycle, and fed with a standard rodent diet and water. The animals were acclimatized to the laboratory for 2 weeks before initiating the studies. Randomization was used when dividing the groups.

#### For acute lung injury

LPS was administered in mice to induce acute inflammatory lung injury, as previously reported [22]. Mice were challenged by intratracheal instillation of LPS (5 mg kg^−1^; dissolved in 0.9% saline) under anesthesia to develop ALI. Mice were randomly divided into four groups: (1) vehicle group (*n* = 7) received 0.9% saline, (2) LPS group (*n* = 7) received 5 mg/kg LPS, (3) PND-1186 group (n = 7) received PND-1186 alone, and (4) LPS + PND group (*n* = 7) received PND-1186 treatment and LPS. PND-1186 was dissolved in Castor oil solution (6% castor oil in PBS) for tail intravenous (i.v.) injection. Mice were i.v. injected with 20 mg/kg PND-1186 30 min before intratracheal instillation of 5 mg/kg LPS. Mice in vehicle group were injected with the vehicle solution and 0.9% saline. After 6 h post-LPS challenge, mice were sacrificed with an overdose of sodium pentobarbital. BALF and lung tissue were collected. The middle lobe of the right lung was collected, and wet weight was recorded. The lung was then heated in a thermostatic oven at 65 °C for 72 h and weighed to determine the baseline lung dry weight. BALF was collected through a tracheal cannula with 1.5 mL saline solution. After centrifugation at 1000 × *g* for 5 min, the supernatant was immediately stored at −80 °C. TNF-α and IL-6 levels were measured. Total proteins were also measured.

#### For septic death

Mice were randomly divided into three groups: (1) vehicle group (*n* = 10) received 0.9% saline and Castor oil solution vehicle, (2) LPS group (*n* = 10) received LPS and Castor oil solution vehicle, and (3) LPS + PND group (*n* = 10) received PND-1186 treatment and LPS. PND-1186 was dissolved in Castor oil solution (6% castor oil in PBS). Male C57BL/6 mice weighing 18–22 g were treated with PND-1186 (20 mg/kg) in a water solution by tail intravenous injection. Approximately 30 min later, an intravenous injection of LPS (25 mg/kg) was administered. Control animals received vehicle alone (Castor oil solution). Body weight changes and survival was recorded for 7 days.

### Lung histology

Lung tissues were fixed in 4% paraformaldehyde, embedded in paraffin, and sectioned at 5-μm thickness. For routine histology, sections were stained with hematoxylin and eosin (H&E).

For tissue immunostaining, sections were deparaffinized in xylene and hydrated using an ethanol gradient. A Pressure-cooker was used for heat-induced antigen retrieval (10 mM sodium citrate buffer, pH 6.5). After incubation with 3% of hydrogen peroxide, all sections were blocked using 5% bovine serum albumin (BSA) and incubated with primary F4/80 antibody (1:100), p-p65 antibody (1:100) overnight at 4 °C. The slides were then incubated with fluorophore-labeled secondary antibody for 10 min. Slides were counterstained with DAPI.

### Statistical analysis

All experiments were randomized and blinded. Data from three independent experiments are expressed as mean ± SEM. The exact sample size (*n*) for each experimental condition is provided and ‘*n*’ refers to independent values, not replicates. Statistical analysis was performed with GraphPad Prism 8.0 software (San Diego, CA, USA). We used one-way ANOVA followed by Dunnett’s post hoc test when comparing more than two groups of data and one-way ANOVA, non-parametric Kruskal–Wallis test, followed by Dunn’s post hoc test when comparing multiple independent groups. *P* values of <0.05 were considered statistically significant. Post-tests were run only if F achieved *P* < 0.05 and there was no significant variance in data homogeneity.

## Results

### LPS activates FAK in macrophages to induce inflammatory cytokines

**Fig. 1:**
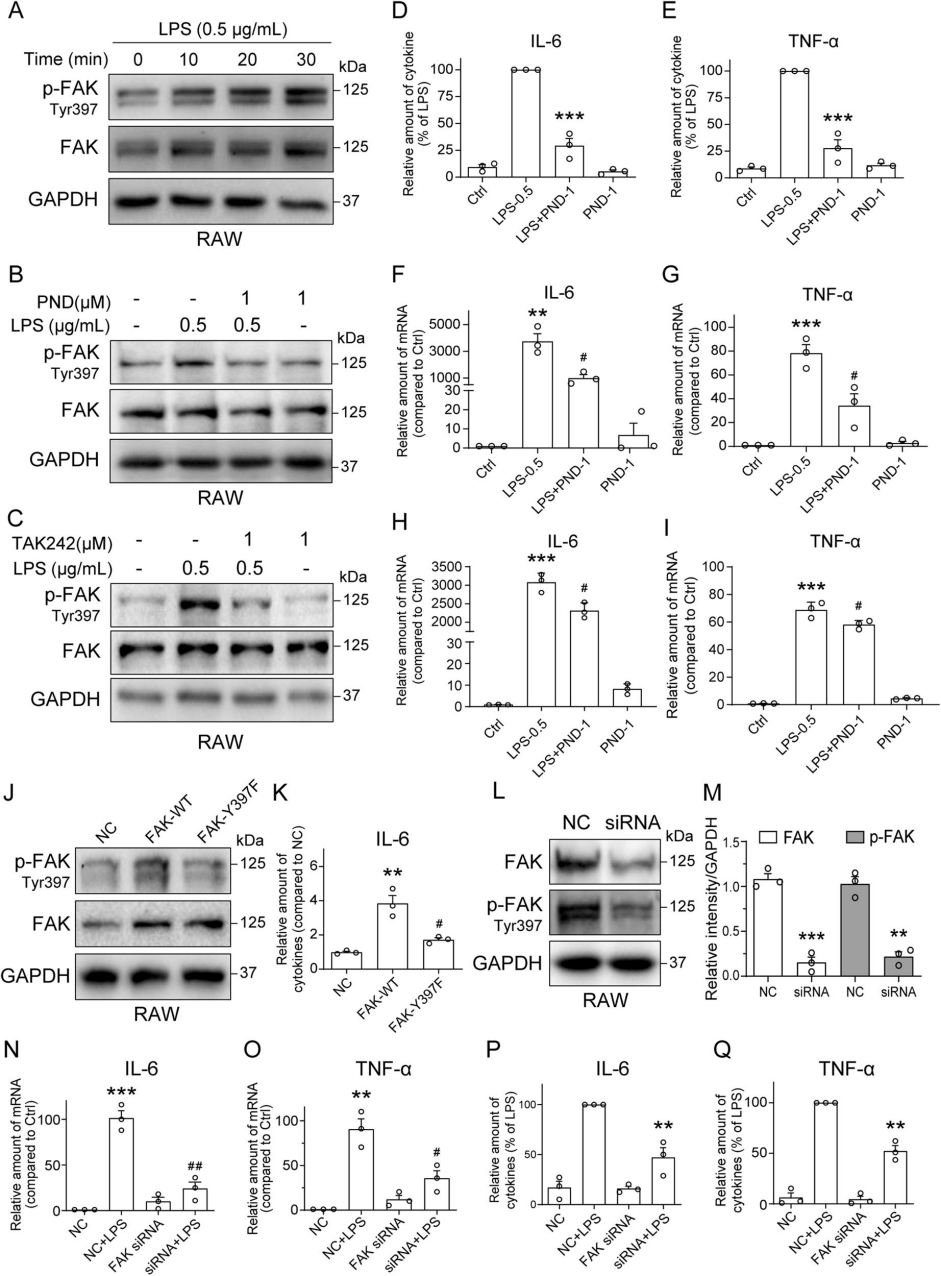
Inhibiting FAK prevents LPS-induced pro-inflammatory responses in macrophages. **A** RAW 264.7 (RAW) macrophage line was exposed to 0.5 µg/mL LPS for the indicated times. Cell lysates were analyzed for p-FAK (Y397) levels. Total FAK and GAPDH used as control. **B**, **C** RAW cells were pretreated with 1 µM FAK inhibitor PND-1186 (PND-1) or 1 µM TLR4 inhibitor TAK242 for 1 h and then exposed to 0.5 μg/mL LPS for 30 min. Cell lysates were probed for p-FAK (Y397) levels. Total FAK and GAPDH were used as control. **D**, **E** RAW cells were pretreated with 1 μM PND-1186 for 1 h and then challenged with 0.5 μg/mL LPS for 24 h. IL-6 (**D**) and TNF-α (**E**) levels in the culture medium were measured by ELISA. Data normalized to total proteins and presented as % LPS [Mean ± SEM, three independent experiments; **P < 0.01 compared to LPS]. **F**, **G** RAW cells were pretreated with 1 μM PND-1186 for 1 h and then exposed to 0.5 μg/mL LPS for 8 h. mRNA levels of IL-6 (**F**) and TNF-α (**G**) were measured by RT-qPCR. Data normalized to β-actin and are expressed as % Ctrl [Mean ± SEM, three independent experiments; **P < 0.01 and ***P < 0.001 compared to Ctrl; ^#^*P* < 0.05 and ^##^*P* < 0.01 compared to LPS]. **H**, **I** RAW cells were treated with 1 μM PND-1186 for 8 h after adding 0.5 μg/mL LPS (1 h). mRNA levels of IL-6 (**H**) and TNF-α (**I**) were measured by RT-qPCR. Data normalized to β-actin and are expressed as % Ctrl [Mean ± SEM, three independent experiments; ***P < 0.001 compared to Ctrl; ^#^*P* < 0.05 compared to LPS]. **J** RAW cells were transfected FAK overexpression plasmid (FAK-WT) and FAK Tyr397 to Phe397 plasmid (FAK-Y397F). The p-FAK^Y397^ and FAK levels were detected by immunoblotting. GAPDH was used as loading control. **K** RAW cells were transfected with FAK-WT or FAK-Y397F plasmid for 36 h. IL-6 levels in culture medium were measured by ELISA. Data normalized to total proteins and presented as fold of NC [Mean ± SEM, three independent experiments; ***P < 0.001 compared to NC; ^##^*P* < 0.01 compared to FAK-WT]. **L** RAW cells were transfected with siRNA against FAK and probed for p-FAK (Y397) levels by immunoblotting. Control cells were transfected with negative control siRNA (NC). **M** Densitometric quantification of FAK and p-FAK (Y397) in RAW cells following siRNA transfection [Mean ± SEM, three independent experiments; **P < 0.01 and ***P < 0.001 compared to NC]. **N**, **O** siRNA transfected RAW cells were exposed to 0.5 μg/mL LPS for 8 h and mRNA levels of IL-6 (**N**) and TNF-α (**O**) were measured by RT-qPCR [Mean ± SEM, three independent experiments; **P < 0.01 compared to LPS]. **P**, **Q** siRNA transfected RAW cells were exposed to 0.5 μg/mL LPS for 24 h and levels of IL-6 (**P**) and TNF-α (**Q**) proteins in the culture medium were measured. Data normalized to total proteins and presented as % Ctrl [Mean ± SEM, 3 independent experiments; **P < 0.01 and ***P < 0.001 compared to Ctrl; ^#^*P* < 0.05 and ^##^*P* < 0.01 compared to LPS].

We first challenged RAW 264.7 macrophage line with 0.5 μg/mL LPS and assessed levels of phosphorylated-FAK (tyrosine 397). Tyrosine 397 phosphorylation of FAK is an essential early step in FAK activation and focal adhesion (dis)assembly [23, 24]. Our results show that LPS causes rapid FAK tyrosine-397 phosphorylation in RAW 264.7 cells (Fig. 1A and Supplementary Fig. 1A). Pretreatment of cells with selective FAK inhibitor PND-1186 (also known as VS-4718) [25] significantly prevented LPS-induced FAK phosphorylation without changing total FAK protein levels (Fig. 1B and Supplementary Fig. 1B). Previous studies have shown that LPS-induced intestinal inflammation through TLR4-FAK-MyD88-IRAK4 signaling pathway [19]. Thus, we examined if LPS activates FAK through TLR4 in RAW 264.7 macrophages. As shown in Fig. 1C and Supplementary Fig. 1C, pretreatment of TLR4 inhibitor TAK242 (1 μM) blocked LPS-induced FAK phosphorylation at Y397 in RAW 264.7 cells, indicating LPS-induced FAK activation is TLR4-dependent. Inhibition of FAK by PND-1186, however, prevented LPS-induced IL-6 and TNF-α protein production and mRNA transcription in RAW cells (Fig. 1D–G). Similar results were observed in THP-1 cells (Supplementary Fig. 1D, E). In addition, we examined the effects of PND-1186 after LPS challenge. We treated with PND-1186 after LPS exposure for 1 h, and the results showed that PND-1186 treatment also suppressed LPS-induced IL-6 and TNF-α mRNA levels (Fig. 1H, I). We performed similar studies in primary mouse macrophages and show LPS-induced FAK phosphorylation (Supplementary Fig. 1F–I). In addition to IL-6 and TNF-α, primary macrophages showed induction of IL-1β and cyclooxygenase-2 (COX2). These proinflammatory factors in primary cells were also suppressed by FAK inhibitor (Supplementary Fig. 1J, K). Pretreatment of TLR4 inhibitor TAK242 also suppressed LPS-induced IL-6 and TNF-α mRNA transcription (Supplementary Fig. 1L, M). To examine the role of FAK tyrosine-397 phosphorylation, we performed site-directed mutation to generate a mouse FAK-Y397F variant (Tyr397 to Phe397). Both wide-type FAK (FAK-WT) plasmid and FAK-Y397F variant were transfected in RAW 264.7 cells (Fig. 1J and Supplementary Fig. 1N). Compared to the negative control (NC) group, overexpression of wide-type FAK (FAK-WT) increased IL-6 protein level, while this increase was not observed in cells transfected with FAK-Y397F variant (Fig. 1K). To further confirm the role of FAK activation in LPS-induced cytokine production, we silenced FAK by siRNA in RAW 264.7 cells (Fig. 1L, M) and observed a complete lack of IL-6 and TNF-α induction by LPS (Fig. 1N–Q). These results show that LPS activates FAK to mediate the expression and production of proinflammatory cytokines in macrophages.

### FAK links LPS to MAPKs and NFκB pathway activation

**Fig. 2:**
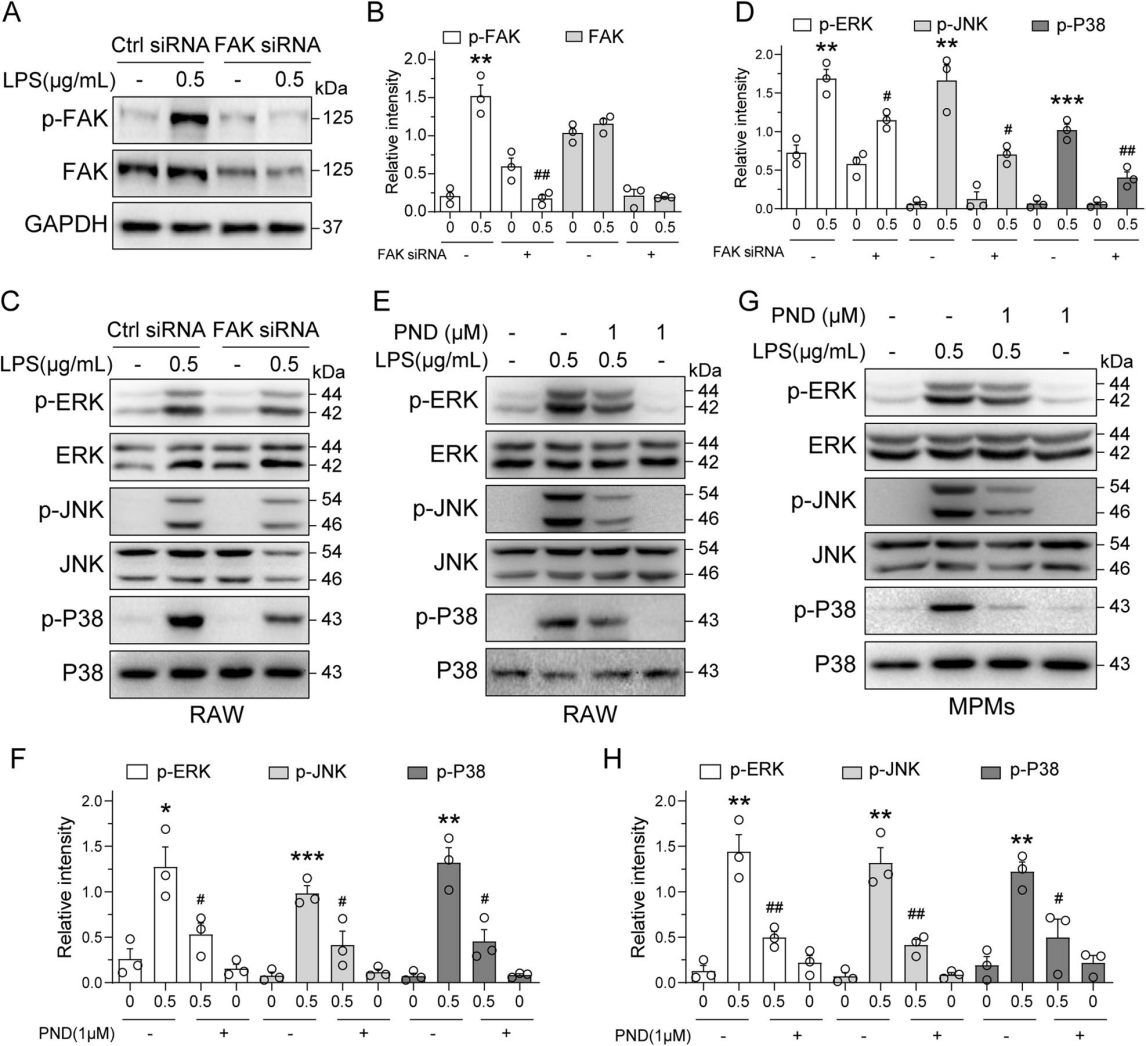
FAK inhibition suppresses LPS-induced MAPK phosphorylation. **A**, **B** RAW 264.7 cells (RAW) were transfected with siRNA against FAK and then exposed to 0.5 μg/mL LPS for 30 min. Control cells were transfected with negative control siRNA (NC). Panel **A** shows immunoblots of p-FAK and FAK. Total FAK and GAPDH were used as controls. Quantification of p-FAK and FAK levels is shown in panel **B** [Mean ± SEM, 3 independent experiments; **P < 0.01 compared to Ctrl siRNA; ^##^*P* < 0.01 compared to Ctrl siRNA+LPS]. **C**, **D** Activation of the MAPK pathway was assessed by measuring phosphorylated ERK, p38, and JNK in panel **C**. Total ERK, p38, and JNK were used as control. Quantification of p-ERK, p-JNK and p-P38 levels is shown in panel **D** [Mean ± SEM, 3 independent experiments; **P < 0.01 and ***P < 0.001 compared to Ctrl siRNA; ^#^*P* < 0.05 and ^##^*P* < 0.01 compared to Ctrl siRNA+LPS]. **E**–**H** Macrophages were pretreated with PND-1186 for 1 h prior to LPS exposure. Figure showing RAW line (**E**) and mouse peritoneal macrophages (MPMs; **G**). Concentration of PND-1186 and LPS are as indicated. Activation of the MAPK pathway was assessed by immunoblotting for phospho-proteins. Densitometric quantification of p-ERK, p-JNK and p-P38 were detected in RAW cells (**F**) and MPMs (**H**) [Mean ± SEM, 3 independent experiments; *P < 0.05, **P < 0.01 and ***P < 0.001 compared to Ctrl; ^#^*P* < 0.05 and ^##^*P* < 0.01 compared to LPS].

MAPKs and NFκB are two main signals in LPS-TLR4 pro-inflammatory cascade. To probe for intracellular signaling proteins which may link FAK to downstream inflammatory responses, we examined the activation of the three arms of MAPKs. We know that enhanced FAK tyrosine phosphorylation links between external signaling and the activation of downstream targets such as the MAPK pathways [26]. LPS increased the phosphorylated protein levels of the ERK, p38 and c-Jun N-terminal kinase (JNK) in RAW 264.7 cells. When we silenced FAK in RAW 264.7 cells, the levels of phospho-MAPK proteins were decreased (Fig. 2A–D). Besides, PND-1186 pretreatment of cells was able to reduce LPS-induced phospho-MAPK proteins (Fig. 2E, F). We confirmed the involvement of MAPKs in primary macrophages isolated from mice by exposing the cells to LPS, with or without pretreatment with PND-1186 (Fig. 2G, H). These data indicated that FAK activated ERK, JNK, and p38.

**Fig. 3:**
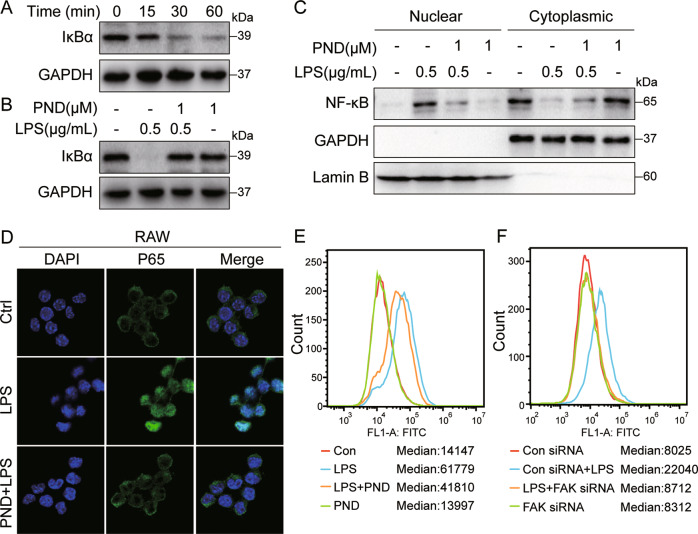
LPS-induced NFκB activation is blocked by FAK inhibitor in macrophages. **A** RAW 264.7 cells were exposed to 0.5 μg/mL LPS for the indicated times. Levels of IκBα were determined as a surrogate of NFκB activity. GAPDH was used as loading control. **B** RAW cells were pretreated with PND-1186 for 1 h before exposure to LPS for 40 min. Levels of IκBα were determined. GAPDH was used as loading control. **C** RAW cells were pretreated with 1 μM PND-1186 for 1 h followed by exposure to 0.5 μg/mL LPS for 1 h. Cytosolic and nuclear p65 levels were determined. GAPDH and Lamin B were used as loading controls. **D** RAW cells were pretreated with 1 μM PND-1186 for 1 h and then stimulated with 0.5 μg/mL LPS for 1 h. Cells were then stained with p65 antibody (green) and counterstained with DAPI (blue). **E** RAW 264.7 cells, transfected with NFκB-RE-EGFP reporter plasmid, were pretreated with 1 μM PND-1186 for 1 h and then exposed to 0.5 μg/mL LPS for 8 h. NFκB activity was detected by flow cytometry. Median fluorescence values are shown. **F** RAW 264.7-NFκB-RE-EGFP cells were transfected with siRNA against FAK and then exposed to 0.5 μg/mL LPS for 8 h. NFκB activity was detected by flow cytometry. Median fluorescence values are shown.

We next determined whether nuclear factor-κB (NFκB) [27] was also involved in this FAK-mediated signaling pathway leading to IL-6 and TNF-α production. To do this, we assessed the levels of inhibitor of κBα (IκBα), a well-established surrogate of NFκB activity. Exposure of RAW 264.7 cells to LPS decreased IκBα levels indicating activation of NFκB (Fig. 3A and Supplementary Fig. 2A). Inhibition of FAK prevented IκBα degradation (Fig. 3B and Supplementary Fig. 2B) suggesting reduced NFκB activation. Similar results were obtained in primary mouse macrophages and THP-1 cells (Supplementary Fig. 2C–H). Western blot analysis of p65 subunit of NFκB in cytosolic and nuclear fractions confirmed LPS-induced activation/translocation of NFκB p65 in the nucleus and reduced translocation in RAW 264.7 cells pretreated with PND-1186 (Fig. 3C). This nuclear translocation of NFκB by LPS was confirmed by staining cells with p65 antibody in both RAW 264.7 cells and primary macrophages (Fig. 3D and Supplementary Fig. 2I). Finally, we transfected RAW 264.7 cells with NFκB reporter plasmid and exposed the cells to LPS, with or without PND-1186 pretreatment. Flow cytometry confirmed that LPS induces NFκB and blocking FAK activation reduces the levels of activated NFκB (Fig. 3E, F).

### FAK-mediated NFκB pathway activation involves FAK-TAK1 interaction

**Fig. 4:**
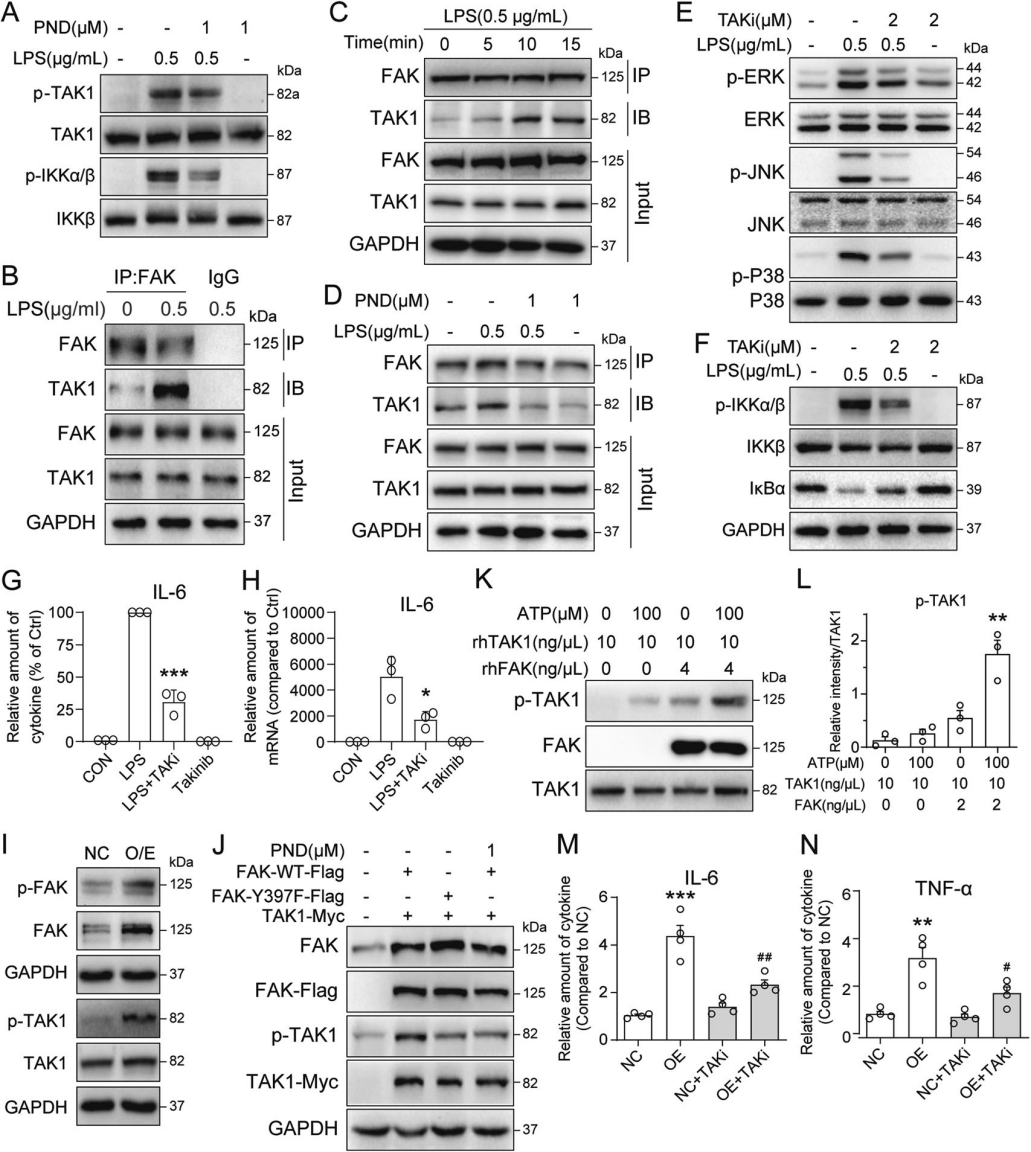
FAK regulates inflammatory responses in macrophages through interacting with TAK1. **A** RAW 264.7 (RAW) cells were pretreated with 1 μM PND-1186 for 1 h and then stimulated with 0.5 μg/mL LPS for 30 min. Cell lysates were analyzed for p-TAK1 (Ser412) and p-IKKα/β (Ser176/180) levels. Total TAK1 and IKKβ were used as control. **B** RAW cells were treated with 0.5 μg/mL LPS for 10 min. Complexes of FAK-TAK1 were detected by immunoprecipitation. **C** RAW cells were treated with 0.5 μg/mL LPS for the indicated times. Complexes of FAK-TAK1 were detected by immunoprecipitation. **D** RAW cells were pretreated with 1 μM PND-1186 for 1 h and then exposed to 0.5 μg/mL LPS for 10 min. Complexes of FAK-TAK1 were detected by immunoprecipitation. **E** RAW cells were pretreated with 2 μM Takinib for 1 h and then exposed to 0.5 μg/mL LPS for 30 min. The phosphorylated ERK, p38 and JNK were examined by western blot assay. Total ERK, p38, and JNK were used as control (TAKi = Takinib). **F** RAW cells were pretreated with 2 μM Takinib for 1 h and then stimulated with 0.5 μg/mL LPS for 30 min. Cell lysates were analyzed for p-IKKα/β and IκBα levels. Total IKKβ and GAPDH were used as control. **G** RAW cells were pretreated with 2 μM Takinib for 1 h and then exposed to 0.5 μg/mL LPS for 24 h. IL-6 proteins in the culture medium were measured by ELISA. Data normalized to total proteins and presented as % LPS [Mean ± SEM, 3 independent experiments; ***P < 0.001 compared to LPS]. **H** RAW cells were pretreated with 2 μM Takinib for 1 h and then exposed to 0.5 μg/mL LPS for 8 h. mRNA levels of IL-6 were measured. Data normalized to β-actin and expressed as % Ctrl [Mean ± SEM, 3 independent experiments; *P < 0.05 compared to LPS]. **I** RAW cells were transfected with FAK-expressing plasmid. After 24 h, levels of p-FAK and p-TAK1 were detected. Total FAK, TAK1, and GAPDH were used as control. Control cells were transfected with negative control/empty vector (NC = negative control, O/E = overexpression). **J** 3T3 cells were transfected with FAK-WT-Flag/FAK-Y397F-Flag/TAK1-Myc expressing plasmid, respectively. After 24 h, levels of p-TAK1 were detected using western blot, with total FAK, Flag, Myc, and GAPDH as controls. **K**, **L** Cell-free kinase assay showing rhFAK phosphorylates rhTAK1. rhTAK1 was incubated with rhFAK in the presence or absence of ATP (100 μM). The samples were separated by SDS-PAGE and western blotting was used to detect p-TAK1, TAK1, and FAK in panel **K**. Densitometric quantification of p-TAK1 levels was determined in panel L [Mean ± SEM, 3 independent experiments; **P < 0.01 compared to rhTAK1]. **M**, **N** FAK-expressing RAW cells were treated with 2 μM Takinib for 12 h. IL-6 (**L**) and TNF-α (**M**) proteins in the culture medium were measured by ELISA. Data normalized to total proteins and presented as fold difference compare to NC [TAKi = Takinib; Mean ± SEM, 3 independent experiments; **P < 0.01 and ***P < 0.001 compared to NC; ^#^*P* < 0.05 and ^##^*P* < 0.01 compared to O/E].

We know that transforming growth factor β-activated kinase 1 (TAK1 or MAP3K7) is an important upstream signaling component of MAPKs in innate immunity [28]. TAK1 can also phosphorylate inhibitor of nuclear factor kappa-B kinase subunit beta (IKKβ) and increase its enzymatic activity [29], which in turn, phosphorylates IκBα and modules NFκB activation. Cells harvested from TAK1-deficient mice show reduced production of inflammatory cytokines and impaired NFκB activation in response to multiple TLR ligands. Therefore, we tested whether FAK-mediated NFκB activation utilizes TAK1. We exposed RAW 264.7, MPMs, and THP-1 cells to LPS and measured the levels of phosphorylated TAK1 (Ser412) and IKKβ (Fig. 4A and Supplementary Fig. 3A–E). Here we show that LPS increases phospho-TAK1 and -IKKβ. However, when cells were pretreated with PND-1186, these increases were not seen. To examine how FAK may relay activation of TAK1, we determined whether FAK directly associates with TAK1 in macrophages. Following exposure of cells to LPS, FAK was immunoprecipitated and levels of associated TAK1 were probed by immunoblotting. Our results show a rapid association of FAK-TAK1 in RAW 264.7 cells following LPS exposure (Fig. 4B, C and Supplementary Fig. 3F–H). Furthermore, such association was significantly dampened in cells that were pretreated with PND-1186 (Fig. 4D, Supplementary Fig. 3I). These data suggest that LPS causes FAK phosphorylation which then leads to TAK1 association and phosphorylation, and downstream signaling. Indeed, treatment of RAW 264.7 cells with selective TAK1 inhibitor Takinib [30] prevented LPS-induced MAPKs phosphorylation (Fig. 4E, Supplementary Fig. 3J). Takinib treatment also inhibits NFκB activation evidenced by decreased IKKβ phosphorylation and IκBα degradation in LPS-challenged RAW 264.7 cells (Fig. 4F, Supplementary Fig. 3K). Further, inhibition of TAK1 by Takinib reduced the protein and mRNA levels of IL-6 by LPS (Fig. 4G, H). We then overexpressed FAK in RAW 264.7 cells, which caused increased basal FAK and phosphorylated-FAK levels (Fig. 4I, Supplementary Fig. 3L). This increase in FAK resulted in TAK1 phosphorylation (Fig. 4J).

To confirm that FAK^Y397^ phosphorylates Ser412 in TAK1, we constructed a mutant FAK^Y397F^, where Y397 was replaced with a phenylalanine. As shown in Fig. 4J, wide-type FAK induced an increase in p-TAK1^Ser412^, which could be inhibited by PND-1186, in the cells transfected with FAK-WT and TAK1-Myc. However, FAK^Y397F^ mutation showed no effect on p-TAK1 in the cells transfected with FAK Y397F and TAK1-Myc. Using recombinant human FAK and TAK1 proteins, we further explored the direct interaction between rhFAK and rhTAK1 at the molecular level. The cell-free kinase phosphorylation assay showed that rhFAK with ATP could directly phosphorylate rhTAK1 at Ser412 site (Fig. 4K, L). Finally, we showed that overexpression of FAK increases IL-6 and TNF-α levels in macrophages (Fig. 4M, N). As expected, inhibition of TAK1 by Takinib was able to reduce the FAK overexpression-increased cytokine levels (Fig. 4M, N). These results indicate the FAK-TAK1-MAPKs/NFκB axle in LPS pro-inflammatory cascade, in which the activated FAK directly phosphorylates TAK1 to activate MAPKs/NFκB and induce the production of proinflammatory cytokines in macrophages.

### Inhibition of FAK protects against LPS-induced inflammatory lung injury and sepsis in mice

**Fig. 5:**
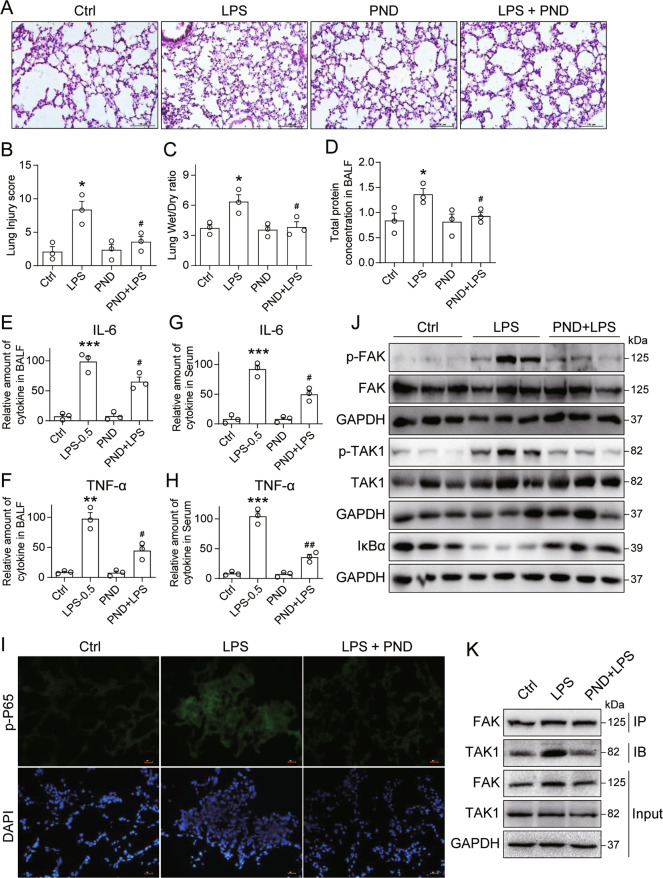
FAK inhibitor protects against LPS-induced inflammatory response in mice. **A** Representative H&E-stained sections of lung tissues harvested from mice following LPS challenge [scale bar = 100 μm]. **B** Lung injury scores were assessed from histological analyses of mouse lung tissues [Mean ± SEM, *n* = 8; *P < 0.05 compared to Ctrl; ^#^*P* < 0.05 compared to LPS]. **C** Lung wet/dry ratio was determined at 6 h after LPS challenge. [Mean ± SEM, *n* = 8; *P < 0.05 compared to Ctrl; ^#^*P* < 0.05 compared to LPS]. **D** BALF was collected 6 h after LPS challenge and the amounts of proteins were measured. [Mean ± SEM, *n* = 8; *P < 0.05 compared to Ctrl; ^#^*P* < 0.05 compared to LPS]. **E**, **F** Levels of IL-6 (**E**) and TNF-α (**F**) in BALF samples. [Mean ± SEM, *n* = 8; **P < 0.01 and ***P < 0.001 compared to Ctrl; ^#^*P* < 0.05 compared to LPS]. **G**, **H** Levels of IL-6 (**G**) and TNF-α (**H**) in serum samples of mice [Mean ± SEM. *N* = 8; ***P < 0.001 compared to Ctrl; ^#^*P* < 0.05 compared to LPS]. **I** Mice were challenged with intratracheal LPS and treated with PND-1186. Lung tissues were stained for inflammation marker p-p65 (green). Slides were counterstained with DAPI (blue) [scale bar = 100 μm]. **J** Protein levels of p-FAK, p-TAK1 and IκBα in mouse lung tissues challenged with intratracheal LPS. Total FAK, TAK, and GAPDH were used as control. **K** Complexes of FAK-TAK1 in mouse lung tissues challenged with intratracheal LPS were detected by immunoprecipitation.

To confirm our findings in an in vivo setting, we challenged mice with intratracheal instillation of LPS and determined whether treatment of mice with PND-1186 affords lung tissue protection. LPS exposure of mice caused significant inflammatory infiltration, structural disturbances, and intra-alveolar hemorrhage, as can be seen in H&E-stained sections (Fig. 5A). These stained lung tissues were used to generate a lung injury score (Fig. 5B). Treatment of mice with PND-1186 appeared to protect mice from LPS-induced lung injury (Fig. 5A, B). The lung wet-to-dry ratio, a marker of pulmonary edema, also indicated that PND-1186 protected mice from LPS injury (Fig. 5C). Total protein concentration in BALF, an indicator of epithelial and cell membrane integrity, was also higher in LPS-challenged mice but comparable to control mice in the PND-1186 group (Fig. 5D). Assessment of IL-6 and TNF-α protein levels in BALF showed significantly reduced levels by PND-1186, however there was no complete normalization (Fig. 5E, F). Similarly, serum levels of these two cytokines showed reduced levels in mice following PND-1186 compared to LPS alone (Fig. 5G, H). Analysis of lung tissues showed increased immunoreactivity to macrophage marker F4/80 as well as the inflammation marker p-P65 (Ser536) in mice challenged with LPS (Fig. 5I and Supplementary Fig. 4C, Supplementary Fig. 4A, B). Lung tissues from PND-1186 treated mice appeared similar to tissues from control mice. These results show that FAK inhibitor prevents LPS-induced lung injury and inflammatory responses. To determine whether similar signaling pathways are at play in vivo, as we determined in our in vitro system, we probed mouse lung tissues by immunoblotting. Here, we show increased phospho-FAK and phospho-TAK1 levels in lung tissues from mice challenged with LPS (Fig. 5J, Supplementary Fig. 4D–F). However, PND-1186 treatment of mice suppressed these increases. Our results also show an interaction of FAK-TAK1 in lung tissues from mice after LPS treatment (Fig. 5K, Supplementary Fig. 4G, H). Furthermore, this association was suppressed when pretreated with PND-1186 in mice.

**Fig. 6:**
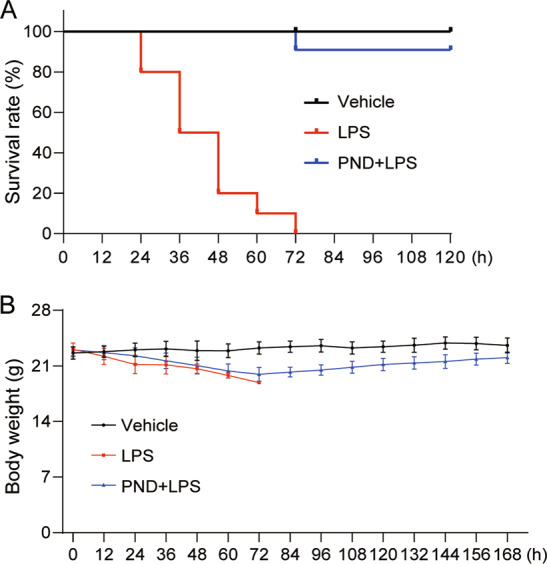
FAK inhibition attenuates LPS-induced septic shock in mice. **A**, **B** PND-1186 treatment enhanced survival in LPS-induced septic mice. C57BL/6 mice (*n* = 10 per group) were injected with 5 mg/kg PND-1186 intravenously, 30 min before an intravenous injection of 25 mg/kg LPS. Survival rates (**A**) and body weight (**B**) were recorded on day 7 following LPS injection.

Finally, we determined whether inhibiting FAK enhances the survival of mice following septic shock. To test this, we challenged mice with 25 mg/kg LPS, with or without intraperitoneal administration of PND-1186. Our results show that PND-1186 significantly increases the survival of mice (Fig. 6A, B). Collectively, our data show that FAK inhibition suppresses TAK1 and NFκB activation, inflammatory factor expression, ALI, and sepsis in LPS-challenged mice.

## Discussion

**Fig. 7:**
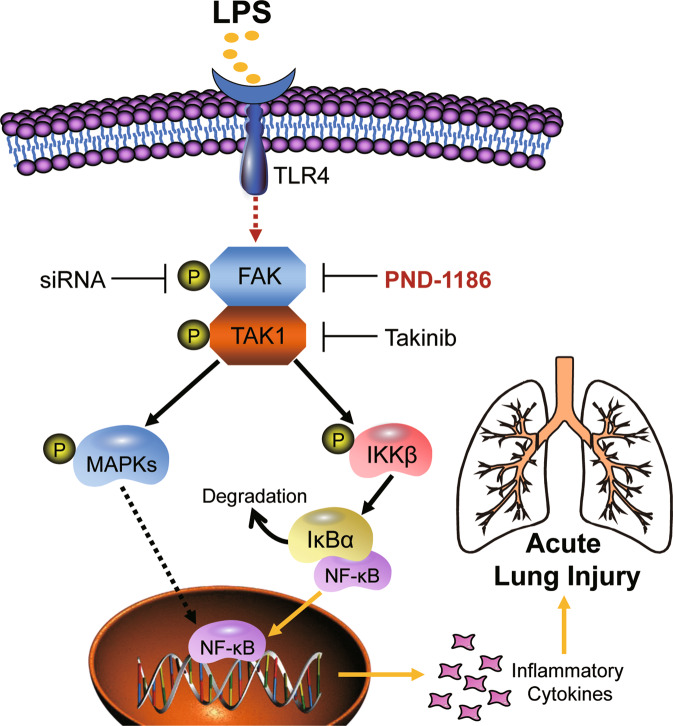
Schematic illustration of the new FAK-TAK1-MAPKs/NFκB signaling axis in LPS-induced inflammatory response in ALI. Upon LPS challenge, activated FAK directly interacts with TAK1 and then phosphorylates TAK1 to activate MAPKs and NFκB signals, which leads to inflammatory cytokine overproduction in both cultured macrophages and lung tissues.

Sepsis is a major cause of ALI and its more severe form, ARDS. The target organ affected first and most severely in intra-abdominal sepsis is the lung [31]. In experimental settings, these injuries are initiated by an endotoxin challenge in mice. This model has been shown to mimic human disease. For example, BAL fluid is known to increase cytokines [32–34], including TNF-α and IL-6, as has been reported in humans. By using this experimental model of bacterial toxin-induced lung injury and macrophage cultures, we identified FAK has a key mediator of inflammatory responses, downstream of LPS. We show that LPS phosphorylates FAK in macrophages. This leads to increased FAK-TAK1 complex formation and induction of NFκB activity and cytokine production. Inhibiting FAK or TAK1 in this system, suppresses MAPKs/NFκB and limits cytokine production in response to LPS. In agreement with these in vitro studies, administration of LPS in mice increased FAK and TAK1 phosphorylation and resulted in increased cytokine levels in serum and BALF. Importantly, treatment of mice with FAK inhibitor was able to surpass this novel inflammatory pathway and prolong survival of mice. Collectively, these results shed new light on the role of FAK in LPS-induced inflammatory ALI (summarized in Fig. 7).

FAK is a non-receptor tyrosine kinase. It associates with the cytoplasmic tail of integrins. Typically, integrin binding to extracellular matrix causes clustering of integrin proteins and FAK autophosphorylation at tyrosine 397. This key phosphorylation induces conformational changes and allows FAK phosphorylation by other kinases. This key phosphorylation induces conformational changes and allows FAK phosphorylation by other kinases. In addition to this prototypical function, recent studies have indicated that LPS may phosphorylate and activate FAK. Exposure of colon adenocarcinoma line Caco-2 to LPS activated FAK and led to increased permeability [19]. In the same study, intraperitoneal administration of LPS increased intestinal permeability through FAK activation. Furthermore, protein I/II, a cell wall component from oral streptococci, has been shown to bind integrin α5β1 and induce the production of IL-6 by human monocytes, epithelial cells, endothelial cells, and fibroblasts [35, 36]. Lastly, embryonic fibroblasts isolated from FAK^−/−^ mice fail to induce IL-6 in response to LPS [37]. These studies suggest that LPS-induced inflammatory lung injury may also entail activation of FAK. Indeed, our studies show that LPS causes a rapid tyrosine-397 phosphorylation of FAK. Inhibition of FAK through a selective inhibitor PND-1186 led to complete normalization of LPS-induced inflammatory cytokine production, including IL-6 in macrophages. In agreement with these studies, we also report that adverse inflammatory responses to LPS in mice are also suppressed by PND-1186 treatment.

TAK1 was initially identified as a regulator of MAPKs, in response to TGF-β and bone morphogenetic protein (BMP) [38]. Investigations then revealed that TAK1 can be activated by many stimuli, including IL-1β, TNF-α, and TLR ligands [39]. Downstream of FAK, myofibroblast differentiation has been shown to occur through TAK1 [40–44]. We also know that TAK1 is an upstream kinase of NFκB. This prompted us to investigate whether FAK links LPS to NFκB through TAK1. In cultured macrophages, LPS-induced TAK1 phosphorylation which could be suppressed by inhibiting FAK. More importantly, our results indicate that LPS enhanced FAK-TAK1 association, as determined by co-immunoprecipitation in macrophage lysates. This association also relied on FAK activation as PND-1186 treatment of cells reduced it.

Although our results indicate rhFAK protein directly phosphorylated rhTAK1^Ser412^ in cell-free system, one important avenue for future studies is to dissect how FAK interacts with TAK1. In addition, we could not completely exclude other kinases involved in FAK-TAK1 interaction in macrophages. For example, studies may involve the identification of obligatory binding proteins linking FAK to TAK1. We know that TAK1 is unique in that its activation requires the formation of complexes with specific binding partner proteins. Some of these known partners include TAK1-binding proteins 1, 2, and 3 (TAB1, TAB2, and TAB3). There are differences in which partners are involved in the activation of TAK1. TAB1 is essential for TAK1 activity and necessary for TGF-β signal transduction [45]. Whereas, TNF-α and IL-1-induced activation of TAK1 requires TAB2 and TAB3 [46]. It is unknown if TABs are involved in FAK-TAK1 interaction. Anyway, our kinase phosphorylation assay using rhFAK and rhTAK1 indicates that, at least, FAK protein is able to directly interact and phosphorylated TAK1.

Another unanswered question is how LPS induces FAK phosphorylation. Although both recent study^19^ and our results show that TLR4 mediated LPS-induced FAK phosphorylation, it remains unclear which TLR4-downstream signaling proteins/kinases are directly involved. LPS stimulates a series of intracellular inflammatory and innate immune signaling. The current data suggest that FAK is mainly in TLR4-MyD88-TAK1-NF-κB signaling pathway, which is consistent with the fact that FAK inhibition or knockdown could not completely reverse LPS-induced up-regulation of inflammatory cytokines (data in Figs. 1 and 3). The precise mechanism by which LPS-induced FAK phosphorylation is certainly a focus of future studies. In addition, this study does not aim to find a stronger anti-inflammatory molecule than TLR4 inhibitor against LPS challenge. As we know, LPS stimulates innate immunity and inflammatory response through directly binding to MD2/TLR4. Therefore, MD2/TLR4 inhibitors must be the strongest molecules against LPS inflammation. However, MD2/TLR4 inhibitors Eritoran and TAK242, unfortunately, both failed in phase 3 clinical trials as anti-sepsis agents [47]. Thus, understanding the signaling mechanism in LPS response, as well as discovering more precise targets, such as FAK, may contribute to develop new and appropriate molecules to treat LPS-induced inflammatory diseases.

In summary, our studies show that FAK inhibition effectively prevents LPS-induced proinflammatory responses in macrophages and in the mouse model of ALI. We have discovered that FAK inhibition afforded an anti-inflammatory function through suppressed TAK1 and MAPKs/NFκB activation. Inhibition of FAK activity has been proposed as a therapeutic target for fibrotic diseases [48–50]. Our studies highlight the value of exploring FAK inhibitors for ALI and sepsis.

## Supplementary information

Reproducibility checklist

Supplementary Data

Original Data File

## Data Availability

All data supporting the findings of this study are available from the corresponding authors on reasonable request.
