# Supplementary material for: FAK mediates LPS-induced inflammatory lung injury through interacting TAK1 and activating TAK1-NFκB pathway
Source: Cell Death Dis. 2022 Jul 8;13(7):589. doi: 10.1038/s41419-022-05046-7 (PMC9270420; doi:10.1038/s41419-022-05046-7)
Supplement: Supplementary file 2 — Supplementary Data [file 41419_2022_5046_MOESM2_ESM.docx]

***Supplementary Information***

**FAK mediates LPS-induced inflammatory lung injury through interacting TAK1 and activating TAK1-NFκB pathway**


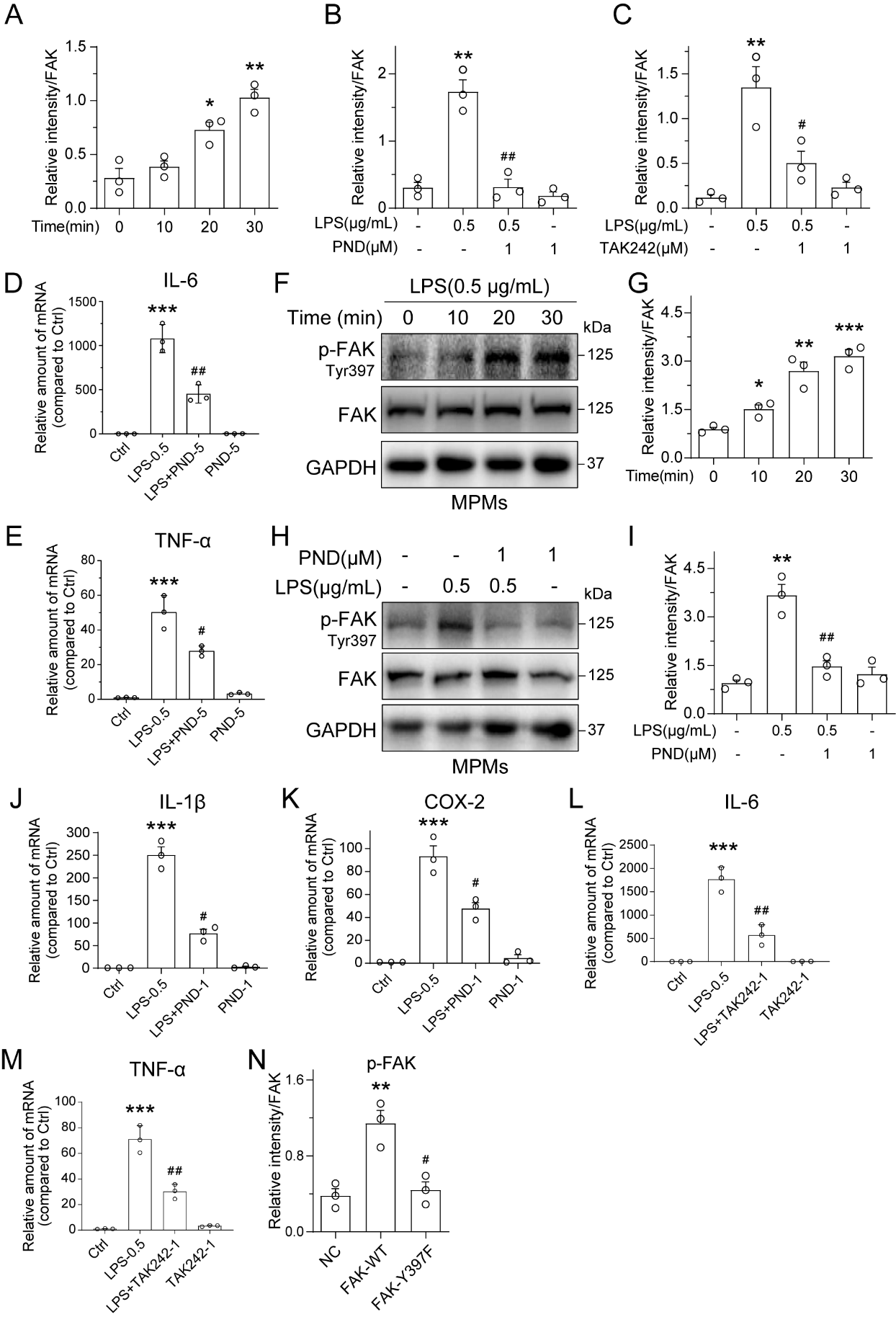


**Supplementary Fig. 1: PND-1186 normalizes LPS-induced inflammatory responses in macrophages.**

**A.** Densitometric quantification of immunoblots shown in Figure 1A [Mean ± SEM, 3 independent experiments; *P<0.05, **P<0.01 compared to 0 min]. **B, C.** Densitometric quantification of immunoblots shown in Figure 1B and 1C [Mean ± SEM, 3 independent experiments; *P<0.05, **P<0.01 compared to Ctrl; ^##^P<0.01 compared to LPS]. **D, E.** THP-1 cells were pretreated with 5 μM PND-1186 for 1 h and then exposed to 0.5 μg/mL LPS for 8 h. mRNA levels of IL-6 (D) and TNF-α (E) were measured by RT-qPCR. Data normalized to β-actin and are expressed as % Ctrl [Mean ± SEM, 3 independent experiments; ***P < 0.001 compared to Ctrl; ^#^P < 0.05 and ^##^P < 0.01 compared to LPS]. **F, G.** Mouse peritoneal macrophages (MPMs) were harvested and exposed to 0.5 μg/mL LPS for the indicated times. Control cells received vehicle alone. Lysates were analyzed for levels of p-FAK (Y397). Panel F shows immunoblots of p-FAK and FAK. Total FAK and GAPDH were used as controls. Quantification of p-FAK levels is shown in panel G [Mean ± SEM, 3 independent experiments; *P < 0.05, **P < 0.01 and ***P < 0.001 compared to 0 min]. **H, I.** MPMs were pretreated with 1 μM PND-1186 for 1 h and then exposed to 0.5 μg/mL LPS for 30 min. Cell lysates were analyzed for p-FAK (Y397) levels. Panel H shows immunoblots of p-FAK and FAK. Total FAK and GAPDH were used as control. Quantification of p-FAK levels is shown in panel I [Mean ± SEM, 3 independent experiments; **P < 0.01 compared to Ctrl; ^##^P < 0.01 compared to LPS]. **J, K.** RAW 264.7 cells (RAW) were pretreated with 1 μM PND-1186 (PND-1) for 1 h and then challenged with 0.5 μg/mL LPS for 8 h. mRNA levels of IL-1β (C) and COX-2 (D) were measured. Data normalized to β-actin and expressed as % Ctrl [Mean ± SEM, 3 independent experiments; **P < 0.01 compared to Ctrl; ^#^P< 0.05 compared to LPS]. **L, M.** RAW cells were pretreated with 1 μM TAK242 for 1 h and then challenged with 0.5 μg/mL LPS for 8 h. mRNA levels of IL-6 (L) and TNF-α (M) were measured. Data normalized to β-actin and expressed as % Ctrl [Mean ± SEM, 3 independent experiments; ***P < 0.001 compared to Ctrl; ^##^P< 0.01 compared to LPS]. **N.** Densitometric quantification of immunoblots shown in Figure 1J [Mean ± SEM, 3 independent experiments; **P < 0.01 compared to NC; ^#^P < 0.05 compared to FAK-WT].


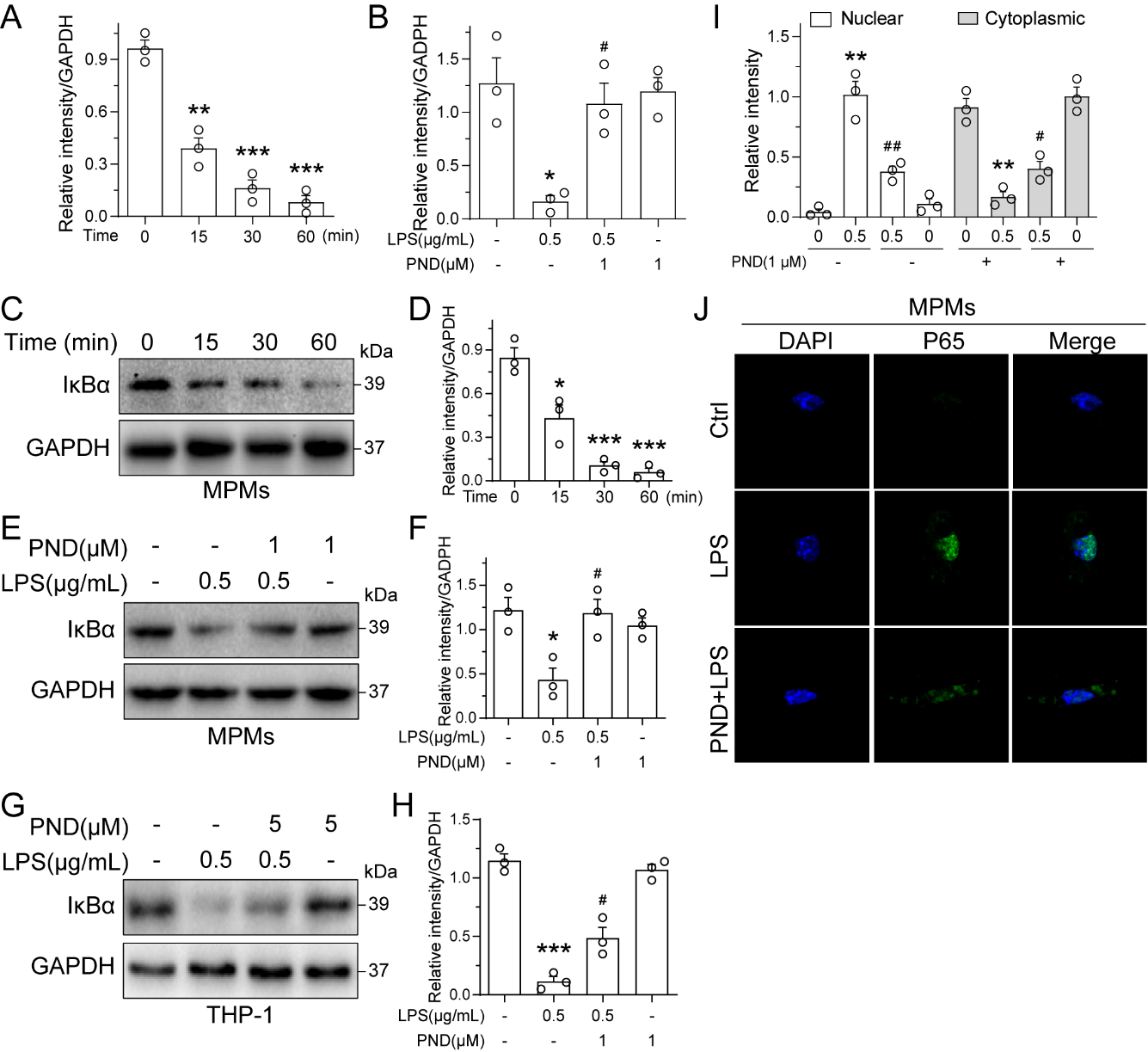


**Supplementary Fig. 2: PND-1186 prevents LPS-induced NF-κB activation in macrophages.**

**A.** Densitometric quantification of immunoblots shown in Figure 3A [Mean ± SEM, 3 independent experiments; **P<0.01 and ***P<0.001 compared to 0 min]. **B.** Densitometric quantification of immunoblots shown in Figure 3B [Mean ± SEM, 3 independent experiments; *P<0.05 compared to Ctrl; ^#^P< 0.05 compared to LPS]. **C, D.** Mouse peritoneal macrophages (MPMs) were challenged with 0.5 μg/mL LPS for the indicated times. Levels of IκBα were determined as a NF-κB activity readout. Panel C shows immunoblots of IκBα. GAPDH was used as loading control. Quantification of IκBα levels is shown in panel D [Mean ± SEM, 3 independent experiments; *P<0.05 and ***P<0.001 compared to 0 min]. **E-H.** MPMs (E) and THP-1 (G) cells were pretreated with 1 μM PND-1186 for 1 h before exposure to 0.5 μg/mL LPS for 40 min. Levels of IκBα were determined. GAPDH was used as loading control. Densitometric quantification of IκBα was detected in MPMs (F) and THP-1 cells (H) [Mean ± SEM, 3 independent experiments; *P < 0.05 and ***P < 0.001 compared to Ctrl; ^#^P< 0.05 compared to LPS]. **I.** Densitometric quantification of immunoblots shown in Figure 3C [Mean ± SEM, 3 independent experiments; **P < 0.01 compared to Ctrl; ^#^P< 0.05 and ^##^P< 0.05 compared to LPS]. **J.** MPMs were pretreated with 1 μM PND-1186 for 1 h, and then stimulated with 0.5 μg/mL LPS for 1 h. Cells were stained with p65 antibody. Counterstaining was performed with DAPI.


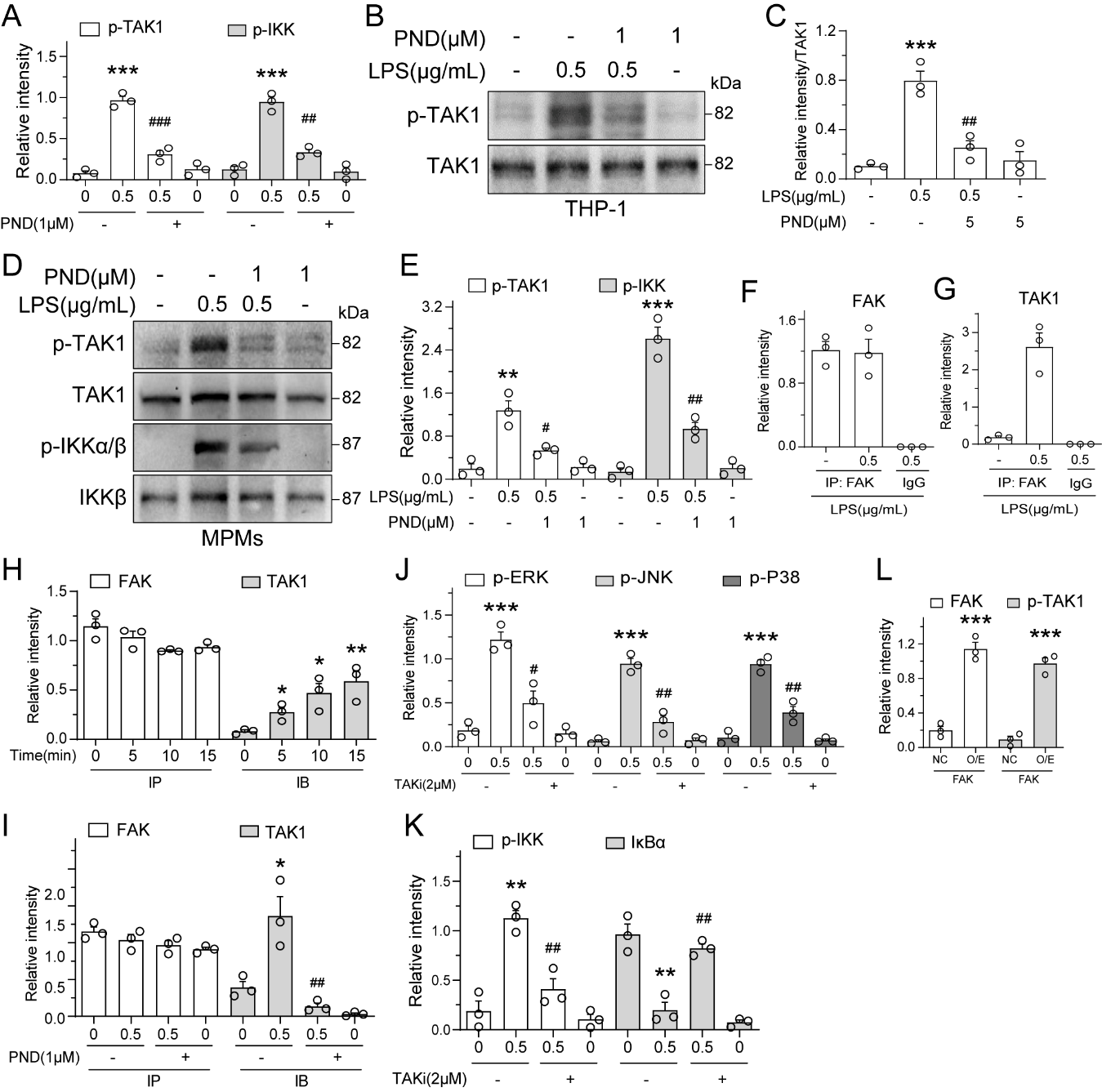


**Supplementary Fig. 3: FAK regulates inflammatory responses in macrophages through interacting with TAK1.**

**A.** Densitometric quantification of immunoblots shown in Figure 4A [***P < 0.001 compared to Ctrl; ^##^P< 0.01 and ^###^P< 0.001 compared to LPS]. **B, C.** THP-1 cells were pretreated with 1 μM PND-1186 for 1 h and then stimulated with 0.5 μg/mL LPS for 30 min. Cell lysates were analyzed for p-TAK1 (Ser412) levels. Panel B shows immunoblots of p-TAK1 (Ser412). Total TAK1 was used as control. Quantification of p-TAK1 levels is shown in panel C [Mean ± SEM, 3 independent experiments; ***P<0.001 compared to Ctrl; ^##^P< 0.01 compared to LPS]. **D, E** MPMs were pretreated with 1 μM PND-1186 for 1 h and then stimulated with 0.5 μg/mL LPS for 30 min. Cell lysates were analyzed for p-TAK1 (Ser412) and p-IKKα/β (Ser176/180) levels. Panel D shows immunoblots of p-TAK1 and p-IKKα/β. Total TAK1 and IKKβ were used as control. Quantification of p-TAK1 and p-IKKα/β levels are shown in panel E [Mean ± SEM, 3 independent experiments; **P<0.01 and ***P<0.001 compared to Ctrl; ^#^P< 0.05 and ^##^P< 0.01 compared to LPS]. **F, G.** Densitometric quantification of immunoblots shown in Figure 4B [Mean ± SEM, 3 independent experiments]. **H.** Densitometric quantification of immunoblots shown in Figure 4C [Mean ± SEM, 3 independent experiments; *P<0.05 and **P<0.01 compared to 0 min]. **I.** Densitometric quantification of immunoblots shown in Figure 4D [Mean ± SEM, 3 independent experiments; *P<0.05 compared to Ctrl; ^#^P< 0.05 compared to LPS]. **J.** Densitometric quantification of immunoblots shown in Figure 4E [Mean ± SEM, 3 independent experiments; ***P<0.001 compared to Ctrl; ^#^P< 0.05 and ^##^P< 0.01 compared to LPS]. **K.** Densitometric quantification of immunoblots shown in Figure 4F [Mean ± SEM, 3 independent experiments; **P<0.01 compared to Ctrl; ^##^P< 0.01 compared to LPS]. **L.** Densitometric quantification of immunoblots shown in Figure 4I [Mean ± SEM, 3 independent experiments; ***P<0.001 compared to NC].


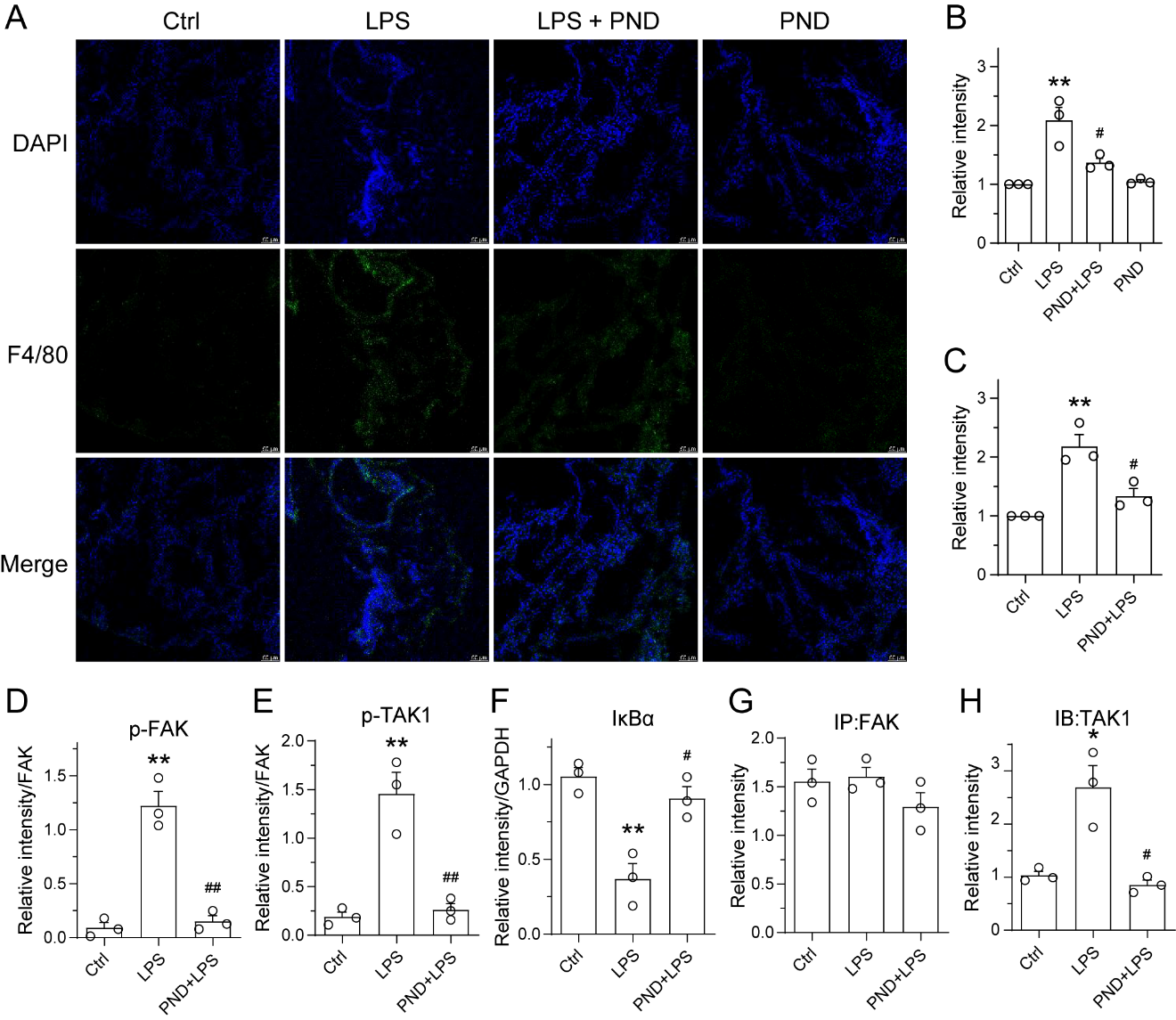


**Supplementary Fig. 4: Macrophage infiltration in lung tissues is inhibited by FAK inhibition.**

**A, B.** Mice were challenged with intratracheal LPS (20 mg/kg) and treated with PND-1186 (5 mg/kg). Lung tissues were stained for macrophage marker F4/80 (green) in panel A. Slides were counterstained with DAPI (blue) [scale bar = 100 μm]. Quantification of macrophage marker F4/80 staining intensity in panel B [Mean ± SEM, 3 independent experiments; **P<0.01 compared to Ctrl; ^#^P< 0.05 compared to LPS]. **C.** Quantification of p-P65 staining intensity shown in Figure 5I [Mean ± SEM, 3 independent experiments; **P<0.01 compared to Ctrl; ^#^P< 0.05 compared to LPS]. **D-F.** Densitometric quantification of immunoblots shown in Figure 5J [Mean ± SEM, 3 independent experiments; **P<0.01 compared to Ctrl; ^#^P< 0.05 and ^##^P< 0.01 compared to LPS]. **G, H.** Densitometric quantification of immunoblots shown in Figure 5K [Mean ± SEM, 3 independent experiments; *P<0.05 compared to Ctrl; ^#^P< 0.05 compared to LPS].

**Table 1.** Primers used in this study.

| Mouse | IL-6_F | GAGGATACCACTCCCAACAGACC |
| --- | --- | --- |
|  | IL-6_R | AAGTGCATCATCGTTGTTCATACA |
|  | IL-1β_F | TCGCAGCAGCACATCAACAAGAG |
|  | IL-1β_R | AGGTCCACGGGAAAGACACAGG |
|  | TNF-α_F | TGATCCGCGACGTGGAA |
|  | TNF-α_R | ACCGCCTGGAGTTCTGGAA |
|  | COX-2_F | TGGTGCCTGGTCTGATGATG |
|  | COX-2_R | GTGGTAACCGCTCAGGTGTTG |
|  | β-actin_F | CCGTGAAAAGATGACCCAGA |
|  | β-actin_R | TACGACCAGAGGCATACAG |
| Human | IL-6_F | ACTCACCTCTTCAGAACGAATTG |
|  | IL-6_R | CCATCTTTGGAAGGTTCAGGTTG |
|  | TNF-α_F | CCTCTCTCTAATCAGCCCTCTG |
|  | TNF-α_R | GCCGATCCACACGGAGTACT |
|  | β-actin_F | CCTGGCACCCAGCACAAT |
|  | β-actin_R | GCCGATCCACACGGAGTACT |
