# Supplementary material for: FAK mediates LPS-induced inflammatory lung injury through interacting TAK1 and activating TAK1-NFκB pathway
Source: Cell Death Dis. 2022 Jul 8;13(7):589. doi: 10.1038/s41419-022-05046-7 (PMC9270420; doi:10.1038/s41419-022-05046-7)
Supplement: Supplementary file 3 — Original Data File [file 41419_2022_5046_MOESM3_ESM.pptx]

## Slide 1
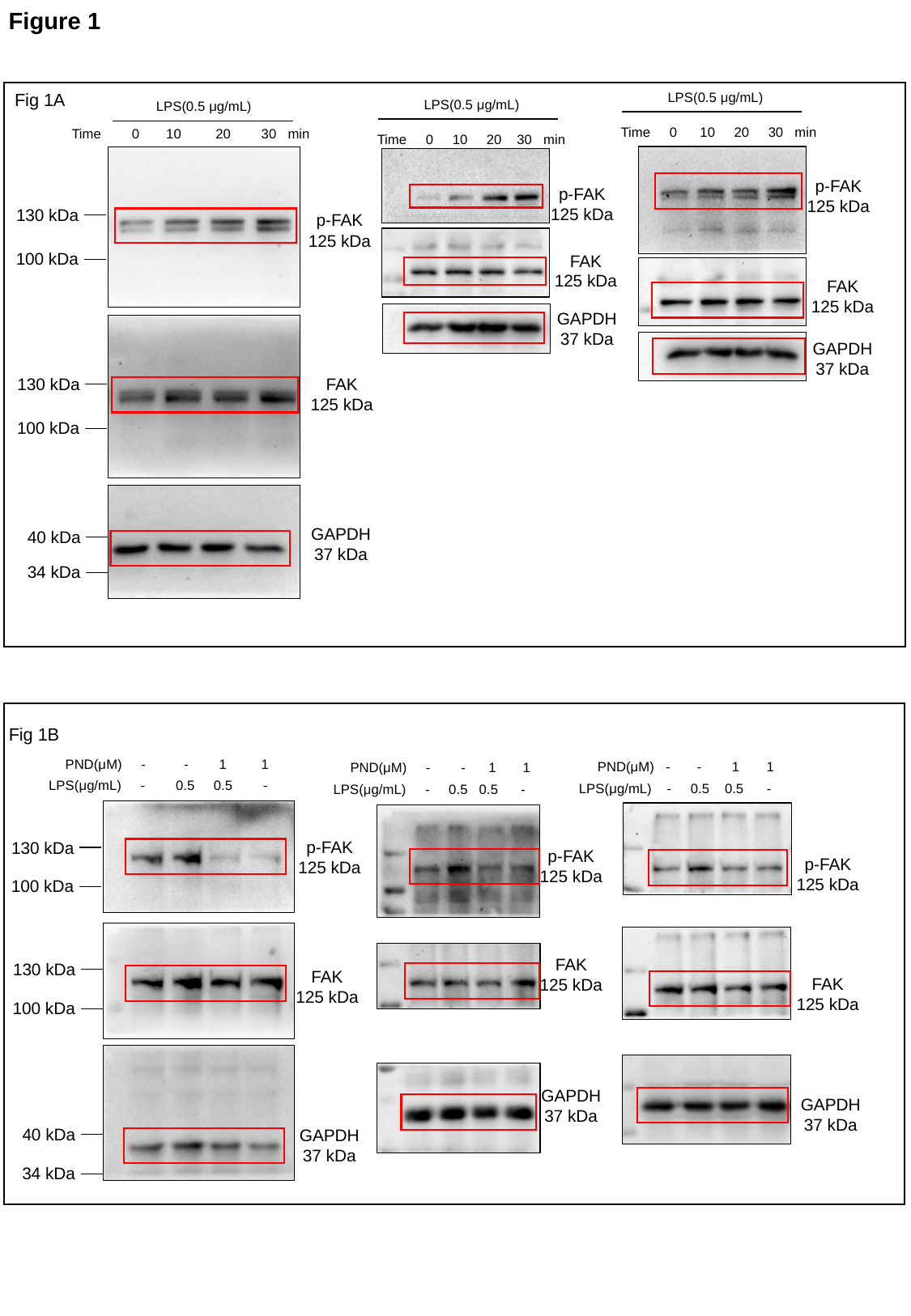

Figure 1
Fig 1A
LPS(0.5 μg/mL)
LPS(0.5 μg/mL)
LPS(0.5 μg/mL)
Time 0 10 20 30 min
Time 0 10 20 30 min
Time 0 10 20 30 min
p-FAK
125 kDa
p-FAK
125 kDa
130 kDa
p-FAK
125 kDa
100 kDa
FAK
125 kDa
FAK
125 kDa
GAPDH
37 kDa
GAPDH
37 kDa
FAK
125 kDa
130 kDa
100 kDa
GAPDH
37 kDa
40 kDa
34 kDa
Fig 1B
 PND(μM) - - 1 1
 PND(μM) - - 1 1
 PND(μM) - - 1 1
LPS(μg/mL) - 0.5 0.5 -
LPS(μg/mL) - 0.5 0.5 -
LPS(μg/mL) - 0.5 0.5 -
p-FAK
125 kDa
130 kDa
p-FAK
125 kDa
p-FAK
125 kDa
100 kDa
FAK
125 kDa
130 kDa
FAK
125 kDa
FAK
125 kDa
100 kDa
GAPDH
37 kDa
GAPDH
37 kDa
40 kDa
GAPDH
37 kDa
34 kDa

## Slide 2
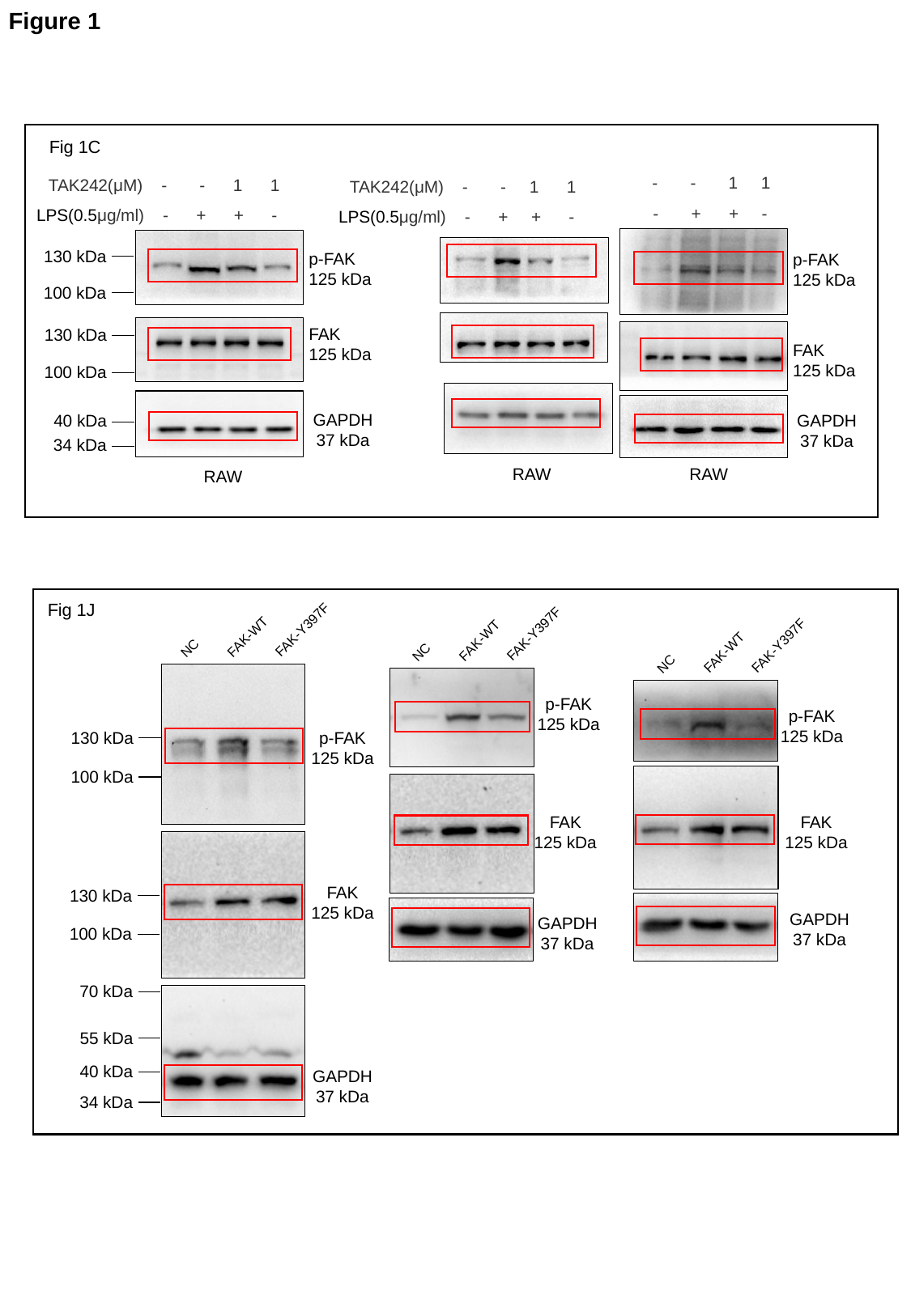

Figure 1
Fig 1C
 - - 1 1
TAK242(μM) - - 1 1
TAK242(μM) - - 1 1
 - + + -
LPS(0.5μg/ml) - + + -
LPS(0.5μg/ml) - + + -
130 kDa
p-FAK
125 kDa
p-FAK
125 kDa
100 kDa
FAK
125 kDa
130 kDa
FAK
125 kDa
100 kDa
GAPDH
37 kDa
40 kDa
GAPDH
37 kDa
34 kDa
RAW
RAW
RAW
Fig 1J
FAK-Y397F
FAK-WT
NC
130 kDa
p-FAK
125 kDa
100 kDa
FAK
125 kDa
130 kDa
100 kDa
70 kDa
55 kDa
40 kDa
GAPDH
37 kDa
34 kDa
FAK-Y397F
FAK-WT
FAK-Y397F
NC
FAK-WT
NC
p-FAK
125 kDa
p-FAK
125 kDa
FAK
125 kDa
FAK
125 kDa
GAPDH
37 kDa
GAPDH
37 kDa

## Slide 3
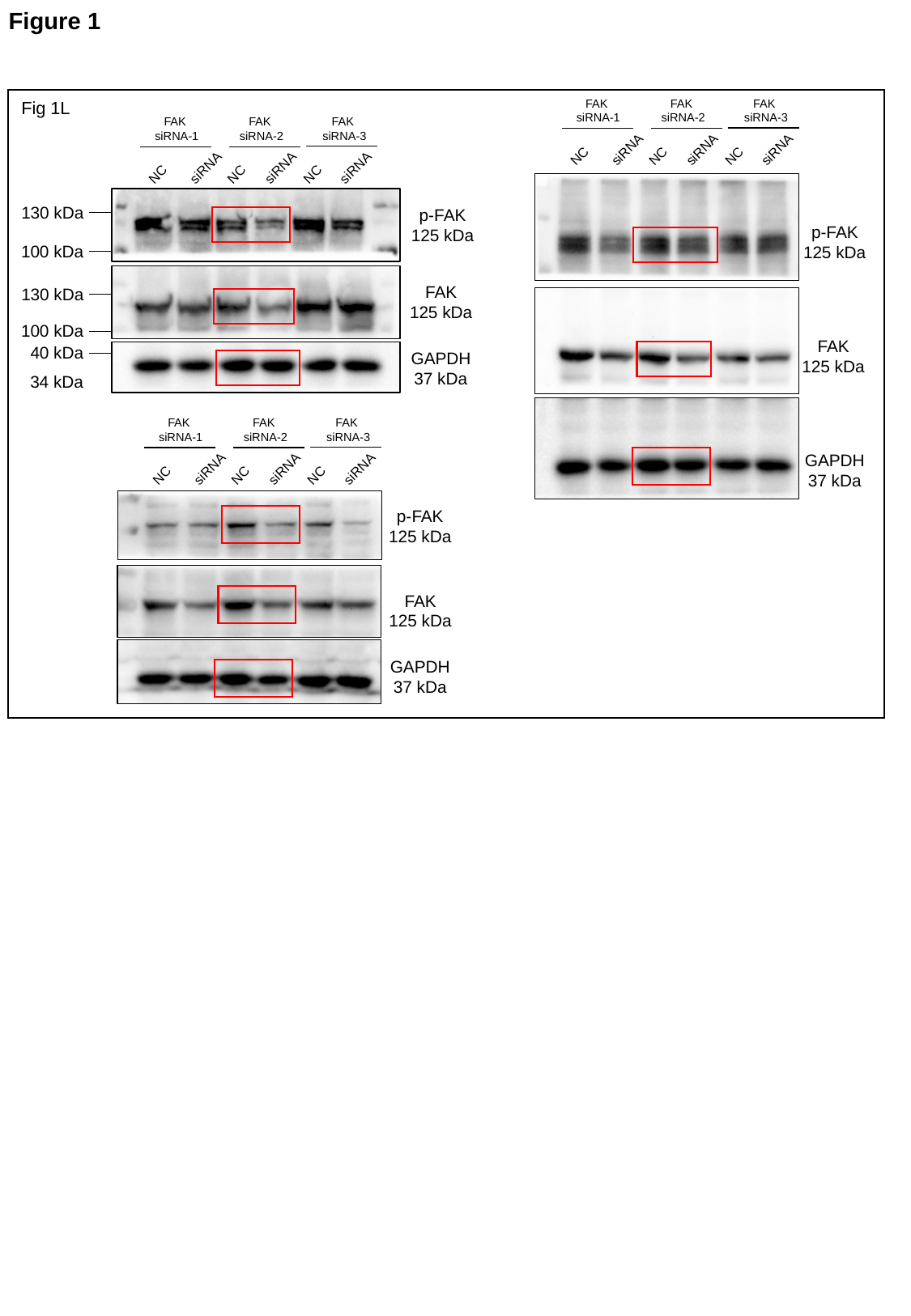

Figure 1
FAK
siRNA-1
FAK
siRNA-2
FAK
siRNA-3
Fig 1L
FAK
siRNA-1
FAK
siRNA-2
FAK
siRNA-3
siRNA
siRNA
siRNA
NC
NC
NC
siRNA
siRNA
siRNA
NC
NC
NC
130 kDa
p-FAK
125 kDa
p-FAK
125 kDa
100 kDa
FAK
125 kDa
130 kDa
100 kDa
FAK
125 kDa
40 kDa
GAPDH
37 kDa
34 kDa
FAK
siRNA-1
FAK
siRNA-2
FAK
siRNA-3
GAPDH
37 kDa
siRNA
siRNA
siRNA
NC
NC
NC
p-FAK
125 kDa
FAK
125 kDa
GAPDH
37 kDa

## Slide 4
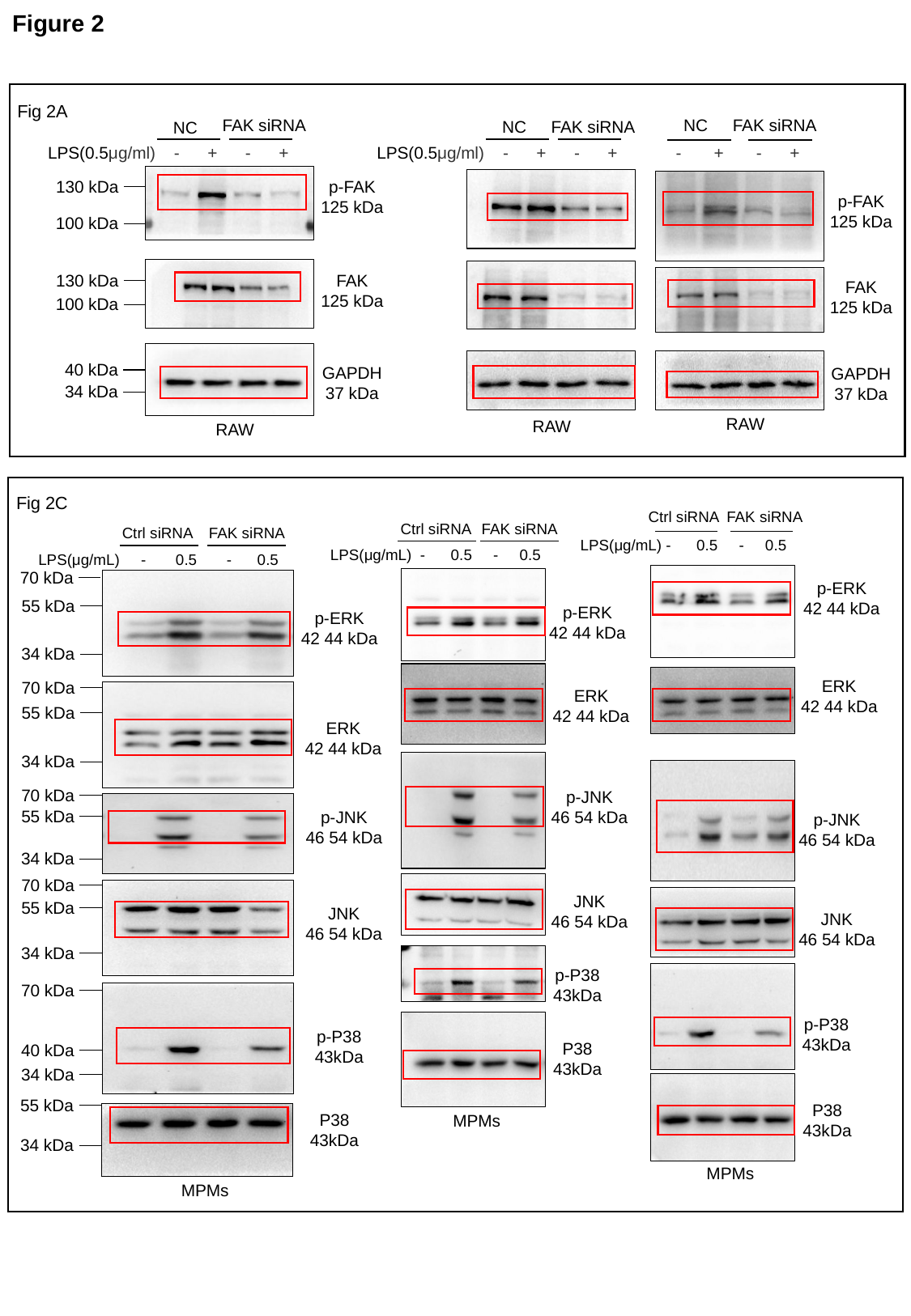

Figure 2
Fig 2A
NC
FAK siRNA
FAK siRNA
NC
FAK siRNA
NC
 - + - +
LPS(0.5μg/ml) - + - +
LPS(0.5μg/ml) - + - +
p-FAK
125 kDa
130 kDa
p-FAK
125 kDa
100 kDa
130 kDa
FAK
125 kDa
FAK
125 kDa
100 kDa
40 kDa
GAPDH
37 kDa
GAPDH
37 kDa
34 kDa
RAW
RAW
RAW
Fig 2C
Ctrl siRNA
FAK siRNA
Ctrl siRNA
FAK siRNA
Ctrl siRNA
FAK siRNA
LPS(μg/mL) - 0.5 - 0.5
LPS(μg/mL) - 0.5 - 0.5
LPS(μg/mL) - 0.5 - 0.5
70 kDa
p-ERK
42 44 kDa
55 kDa
p-ERK
42 44 kDa
p-ERK
42 44 kDa
34 kDa
ERK
42 44 kDa
70 kDa
ERK
42 44 kDa
55 kDa
ERK
42 44 kDa
34 kDa
70 kDa
p-JNK
46 54 kDa
55 kDa
p-JNK
46 54 kDa
p-JNK
46 54 kDa
34 kDa
70 kDa
JNK
46 54 kDa
55 kDa
JNK
46 54 kDa
JNK
46 54 kDa
34 kDa
p-P38
43kDa
70 kDa
p-P38
43kDa
p-P38
43kDa
P38
43kDa
40 kDa
34 kDa
55 kDa
P38
43kDa
P38
43kDa
MPMs
34 kDa
MPMs
MPMs

## Slide 5
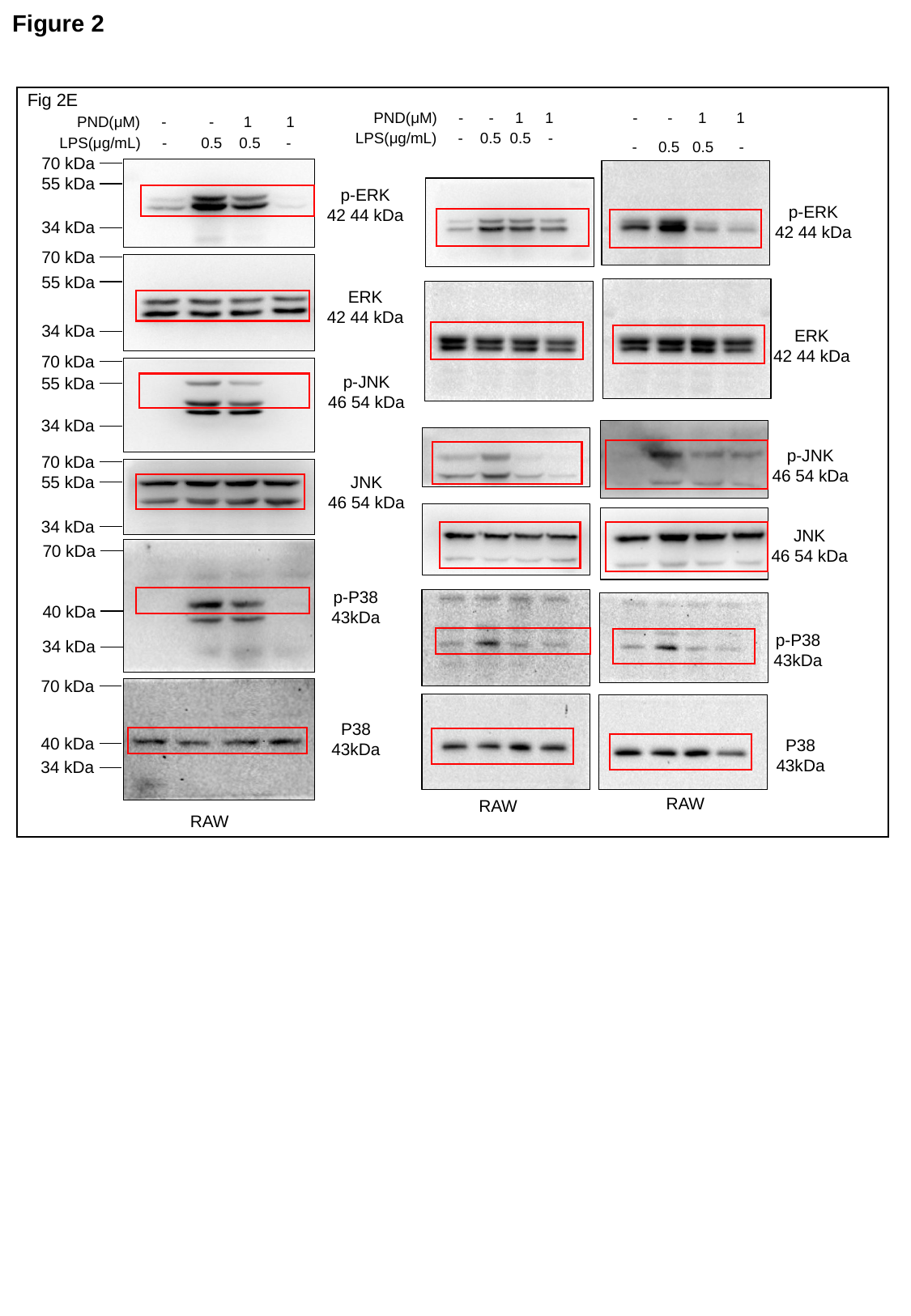

Figure 2
Fig 2E
 PND(μM) - - 1 1
LPS(μg/mL) - 0.5 0.5 -
70 kDa
55 kDa
p-ERK
42 44 kDa
34 kDa
70 kDa
55 kDa
ERK
42 44 kDa
34 kDa
70 kDa
p-JNK
46 54 kDa
55 kDa
34 kDa
70 kDa
JNK
46 54 kDa
55 kDa
34 kDa
70 kDa
p-P38
43kDa
40 kDa
34 kDa
70 kDa
P38
43kDa
40 kDa
34 kDa
 PND(μM) - - 1 1
 - - 1 1
LPS(μg/mL) - 0.5 0.5 -
 - 0.5 0.5 -
p-ERK
42 44 kDa
ERK
42 44 kDa
p-JNK
46 54 kDa
JNK
46 54 kDa
p-P38
43kDa
P38
43kDa
RAW
RAW
RAW

## Slide 6
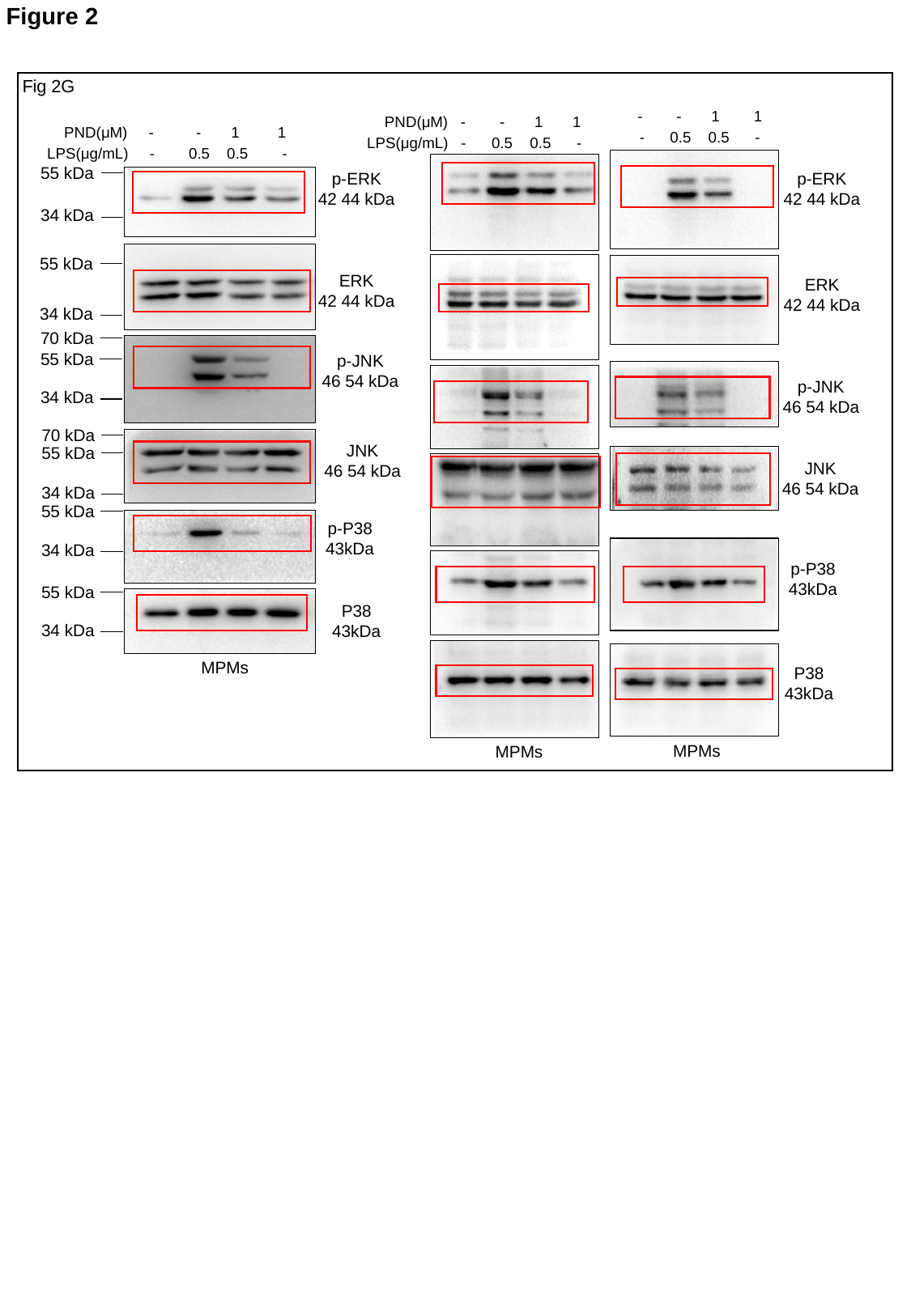

Figure 2
Fig 2G
- - 1 1
 PND(μM) - - 1 1
 PND(μM) - - 1 1
 - 0.5 0.5 -
LPS(μg/mL) - 0.5 0.5 -
LPS(μg/mL) - 0.5 0.5 -
55 kDa
p-ERK
42 44 kDa
p-ERK
42 44 kDa
34 kDa
55 kDa
ERK
42 44 kDa
ERK
42 44 kDa
34 kDa
70 kDa
55 kDa
p-JNK
46 54 kDa
p-JNK
46 54 kDa
34 kDa
70 kDa
JNK
46 54 kDa
55 kDa
JNK
46 54 kDa
34 kDa
55 kDa
p-P38
43kDa
34 kDa
p-P38
43kDa
55 kDa
P38
43kDa
34 kDa
MPMs
P38
43kDa
MPMs
MPMs

## Slide 7
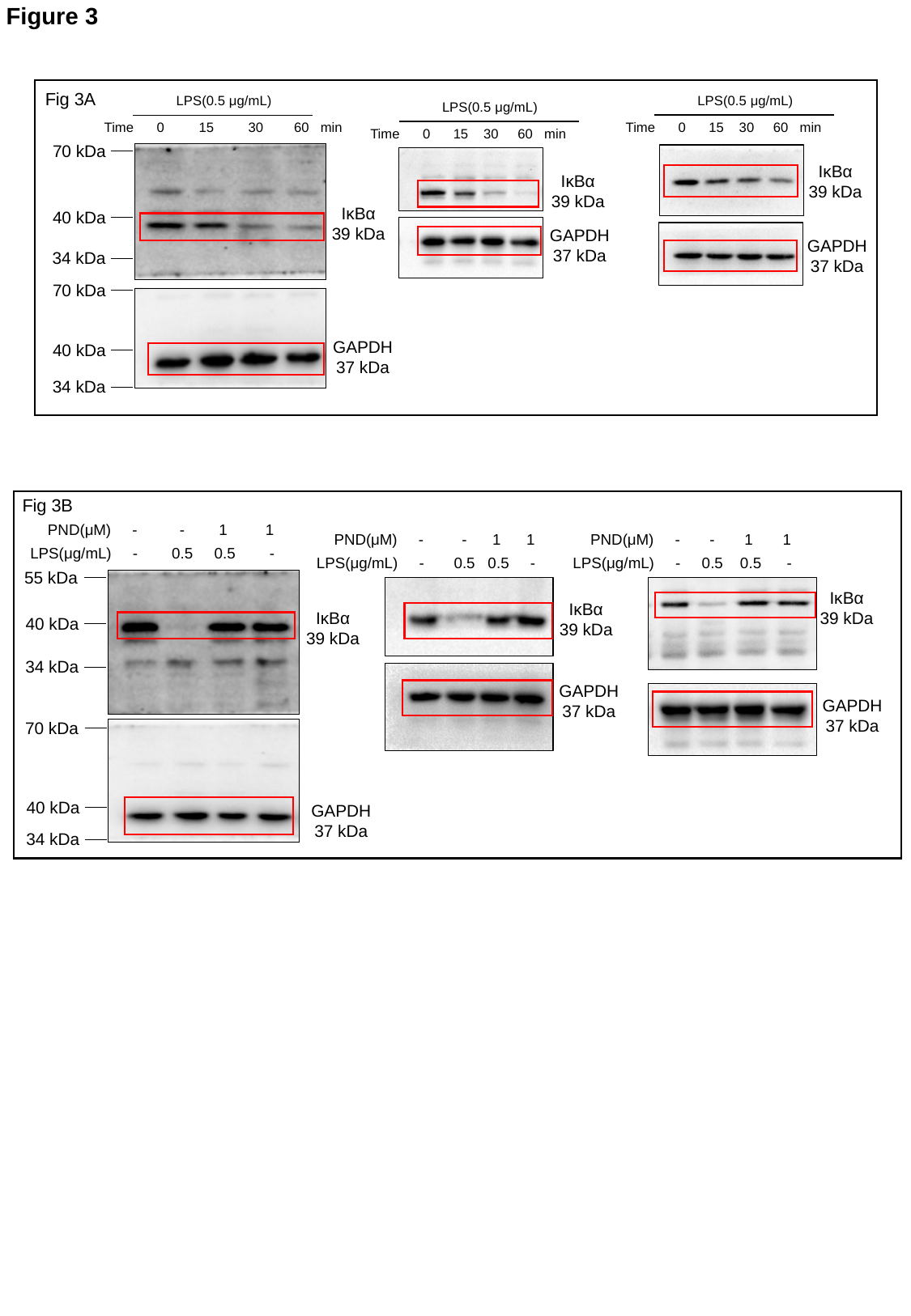

Figure 3
Fig 3A
LPS(0.5 μg/mL)
LPS(0.5 μg/mL)
LPS(0.5 μg/mL)
Time 0 15 30 60 min
Time 0 15 30 60 min
Time 0 15 30 60 min
70 kDa
IκBα
39 kDa
IκBα
39 kDa
IκBα
39 kDa
40 kDa
GAPDH
37 kDa
GAPDH
37 kDa
34 kDa
70 kDa
GAPDH
37 kDa
40 kDa
34 kDa
Fig 3B
 PND(μM) - - 1 1
 PND(μM) - - 1 1
 PND(μM) - - 1 1
LPS(μg/mL) - 0.5 0.5 -
LPS(μg/mL) - 0.5 0.5 -
LPS(μg/mL) - 0.5 0.5 -
55 kDa
IκBα
39 kDa
IκBα
39 kDa
IκBα
39 kDa
40 kDa
34 kDa
GAPDH
37 kDa
GAPDH
37 kDa
70 kDa
40 kDa
GAPDH
37 kDa
34 kDa

## Slide 8
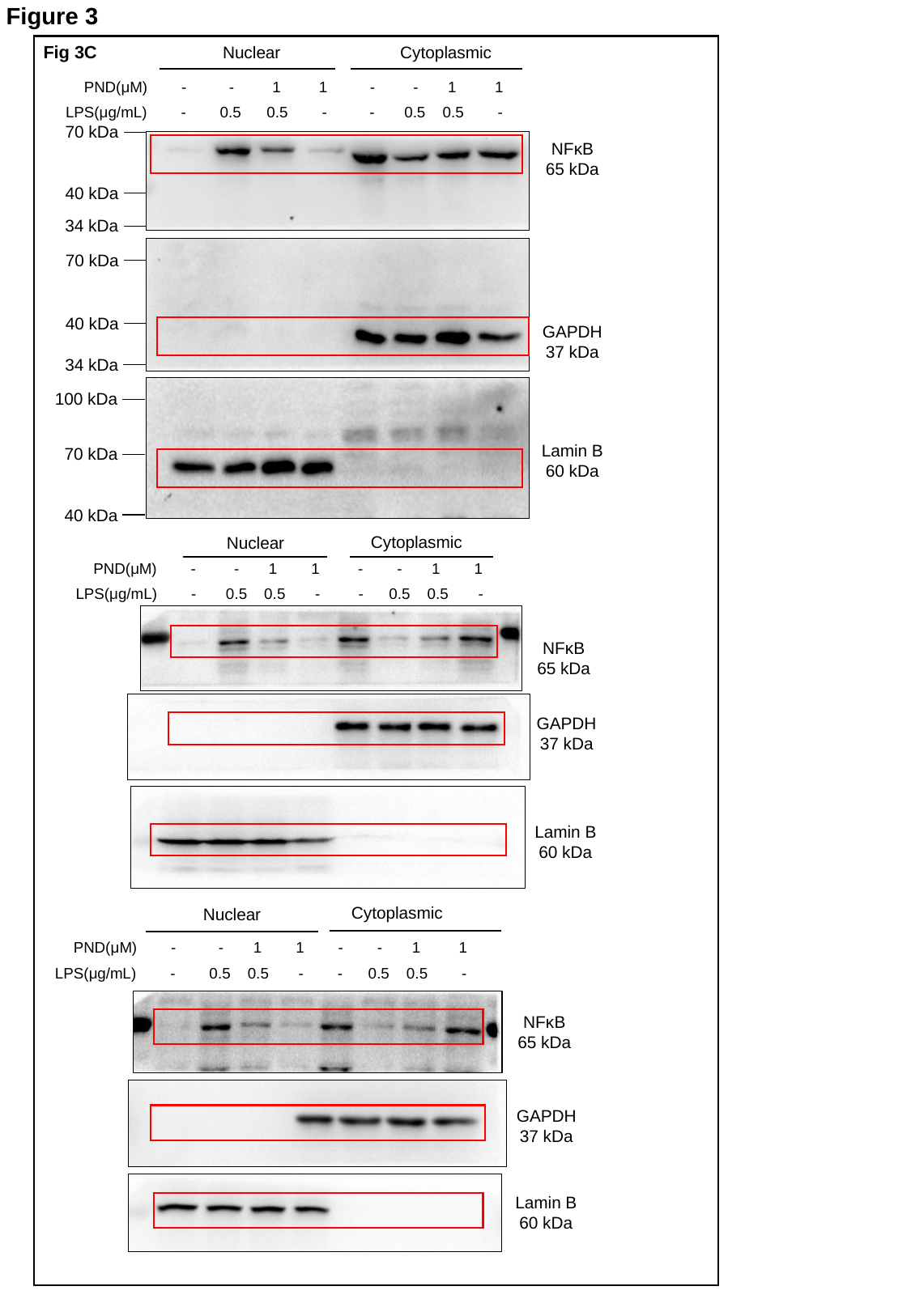

Figure 3
Fig 3C
Nuclear
Cytoplasmic
 PND(μM) - - 1 1 - - 1 1
LPS(μg/mL) - 0.5 0.5 - - 0.5 0.5 -
70 kDa
NFκB
65 kDa
40 kDa
34 kDa
70 kDa
40 kDa
GAPDH
37 kDa
34 kDa
100 kDa
Lamin B
60 kDa
70 kDa
40 kDa
Cytoplasmic
Nuclear
 PND(μM) - - 1 1 - - 1 1
LPS(μg/mL) - 0.5 0.5 - - 0.5 0.5 -
NFκB
65 kDa
GAPDH
37 kDa
Lamin B
60 kDa
Cytoplasmic
Nuclear
 PND(μM) - - 1 1 - - 1 1
LPS(μg/mL) - 0.5 0.5 - - 0.5 0.5 -
NFκB
65 kDa
GAPDH
37 kDa
Lamin B
60 kDa

## Slide 9
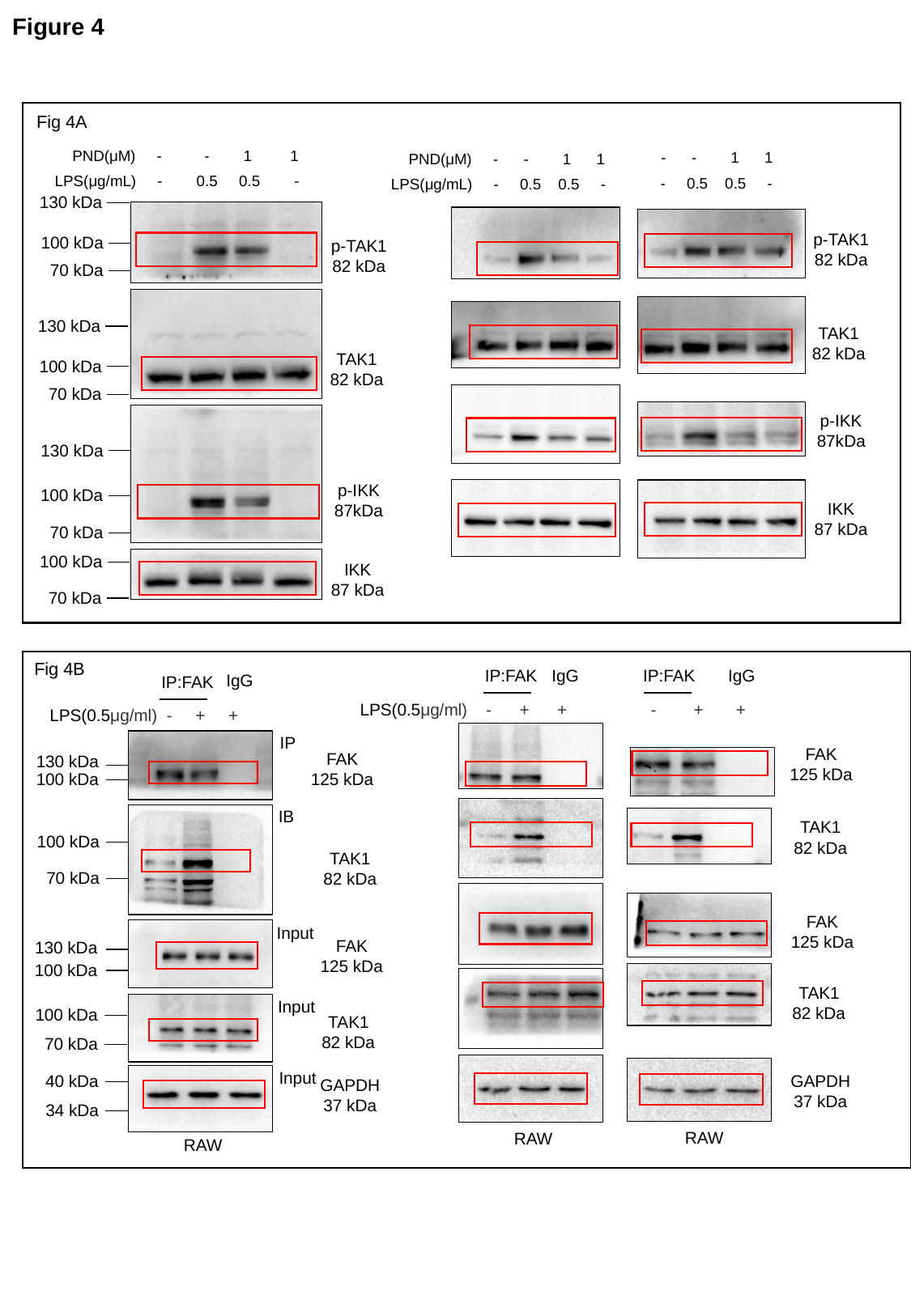

Figure 4
Fig 4A
 PND(μM) - - 1 1
 - - 1 1
 PND(μM) - - 1 1
LPS(μg/mL) - 0.5 0.5 -
 - 0.5 0.5 -
LPS(μg/mL) - 0.5 0.5 -
130 kDa
p-TAK1
82 kDa
100 kDa
p-TAK1
82 kDa
70 kDa
130 kDa
TAK1
82 kDa
TAK1
82 kDa
100 kDa
70 kDa
p-IKK
87kDa
130 kDa
p-IKK
87kDa
100 kDa
IKK
87 kDa
70 kDa
100 kDa
IKK
87 kDa
70 kDa
Fig 4B
IP:FAK
IgG
IP:FAK
IgG
IgG
IP:FAK
LPS(0.5μg/ml) - + +
- + +
LPS(0.5μg/ml) - + +
IP
FAK
125 kDa
FAK
125 kDa
130 kDa
100 kDa
IB
TAK1
82 kDa
100 kDa
TAK1
82 kDa
70 kDa
FAK
125 kDa
Input
FAK
125 kDa
130 kDa
100 kDa
TAK1
82 kDa
Input
100 kDa
TAK1
82 kDa
70 kDa
Input
40 kDa
GAPDH
37 kDa
GAPDH
37 kDa
34 kDa
RAW
RAW
RAW

## Slide 10
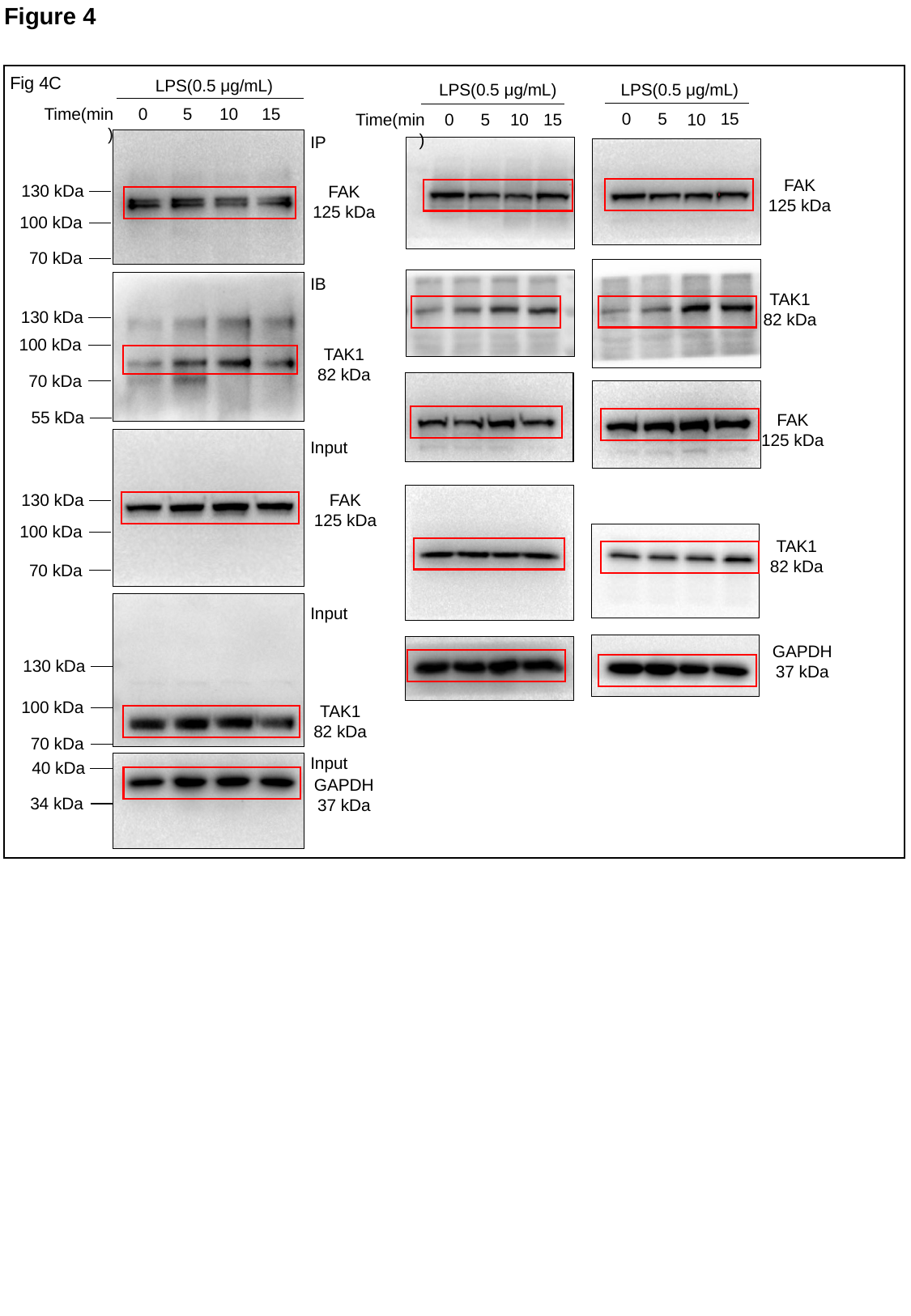

Figure 4
Fig 4C
LPS(0.5 μg/mL)
LPS(0.5 μg/mL)
LPS(0.5 μg/mL)
Time(min)
5
15
0
10
5
15
0
10
Time(min)
5
15
0
10
IP
FAK
125 kDa
130 kDa
FAK
125 kDa
100 kDa
70 kDa
IB
TAK1
82 kDa
130 kDa
100 kDa
TAK1
82 kDa
70 kDa
55 kDa
FAK
125 kDa
Input
FAK
125 kDa
130 kDa
100 kDa
TAK1
82 kDa
70 kDa
Input
GAPDH
37 kDa
130 kDa
100 kDa
TAK1
82 kDa
70 kDa
Input
40 kDa
GAPDH
37 kDa
34 kDa

## Slide 11
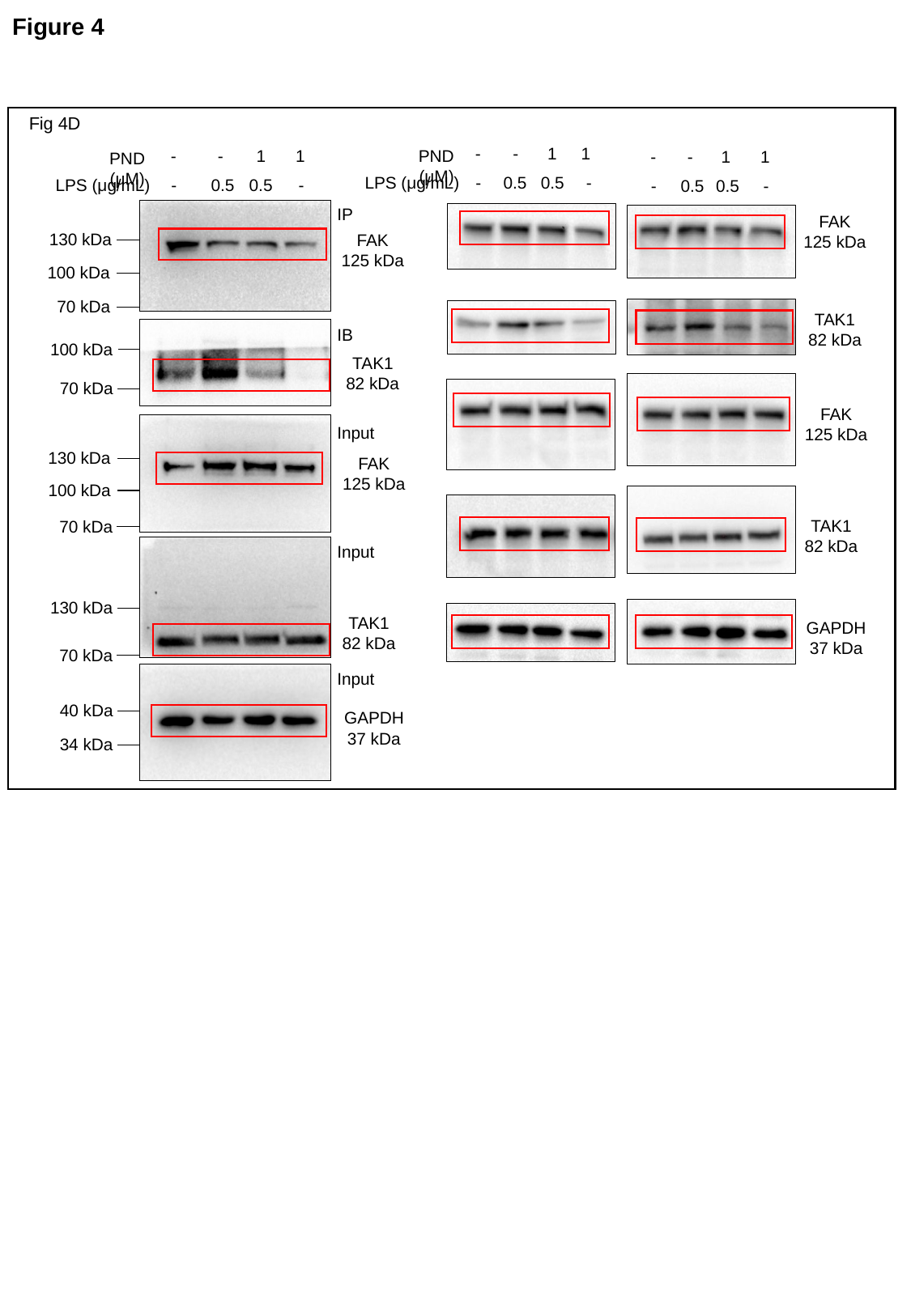

Figure 4
Fig 4D
-
-
1
1
-
-
1
1
PND (μM)
-
-
1
1
PND (μM)
LPS (μg/mL)
-
0.5
0.5
-
LPS (μg/mL)
-
0.5
0.5
-
-
0.5
0.5
-
IP
FAK
125 kDa
130 kDa
FAK
125 kDa
100 kDa
70 kDa
TAK1
82 kDa
IB
100 kDa
TAK1
82 kDa
70 kDa
FAK
125 kDa
Input
130 kDa
FAK
125 kDa
100 kDa
TAK1
82 kDa
70 kDa
Input
130 kDa
TAK1
82 kDa
GAPDH
37 kDa
70 kDa
Input
40 kDa
GAPDH
37 kDa
34 kDa

## Slide 12
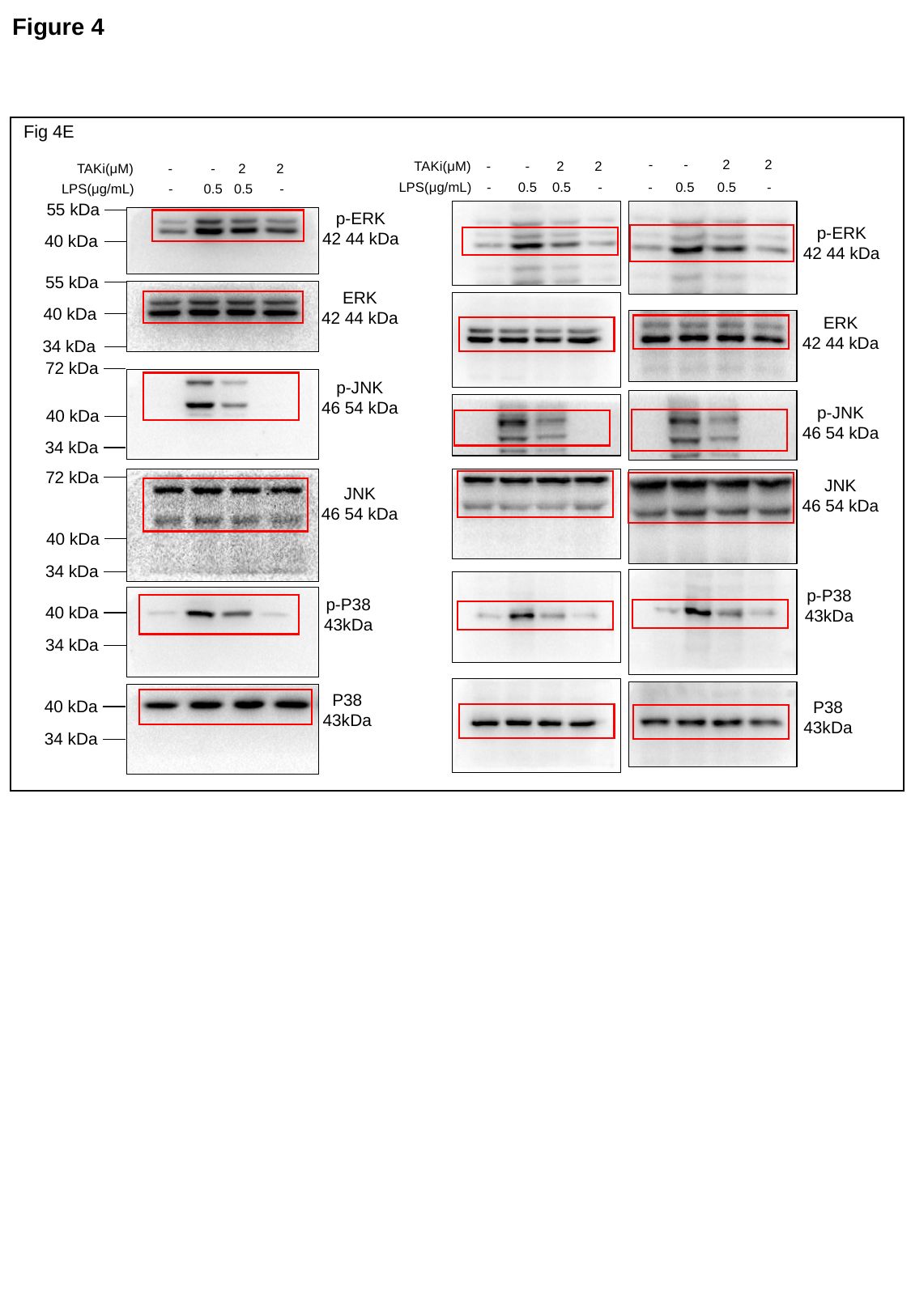

Figure 4
Fig 4E
- - 2 2
 TAKi(μM) - - 2 2
 TAKi(μM) - - 2 2
LPS(μg/mL) - 0.5 0.5 -
- 0.5 0.5 -
LPS(μg/mL) - 0.5 0.5 -
55 kDa
p-ERK
42 44 kDa
p-ERK
42 44 kDa
40 kDa
55 kDa
ERK
42 44 kDa
40 kDa
ERK
42 44 kDa
34 kDa
72 kDa
p-JNK
46 54 kDa
p-JNK
46 54 kDa
40 kDa
34 kDa
72 kDa
JNK
46 54 kDa
JNK
46 54 kDa
40 kDa
34 kDa
p-P38
43kDa
p-P38
43kDa
40 kDa
34 kDa
P38
43kDa
40 kDa
P38
43kDa
34 kDa

## Slide 13
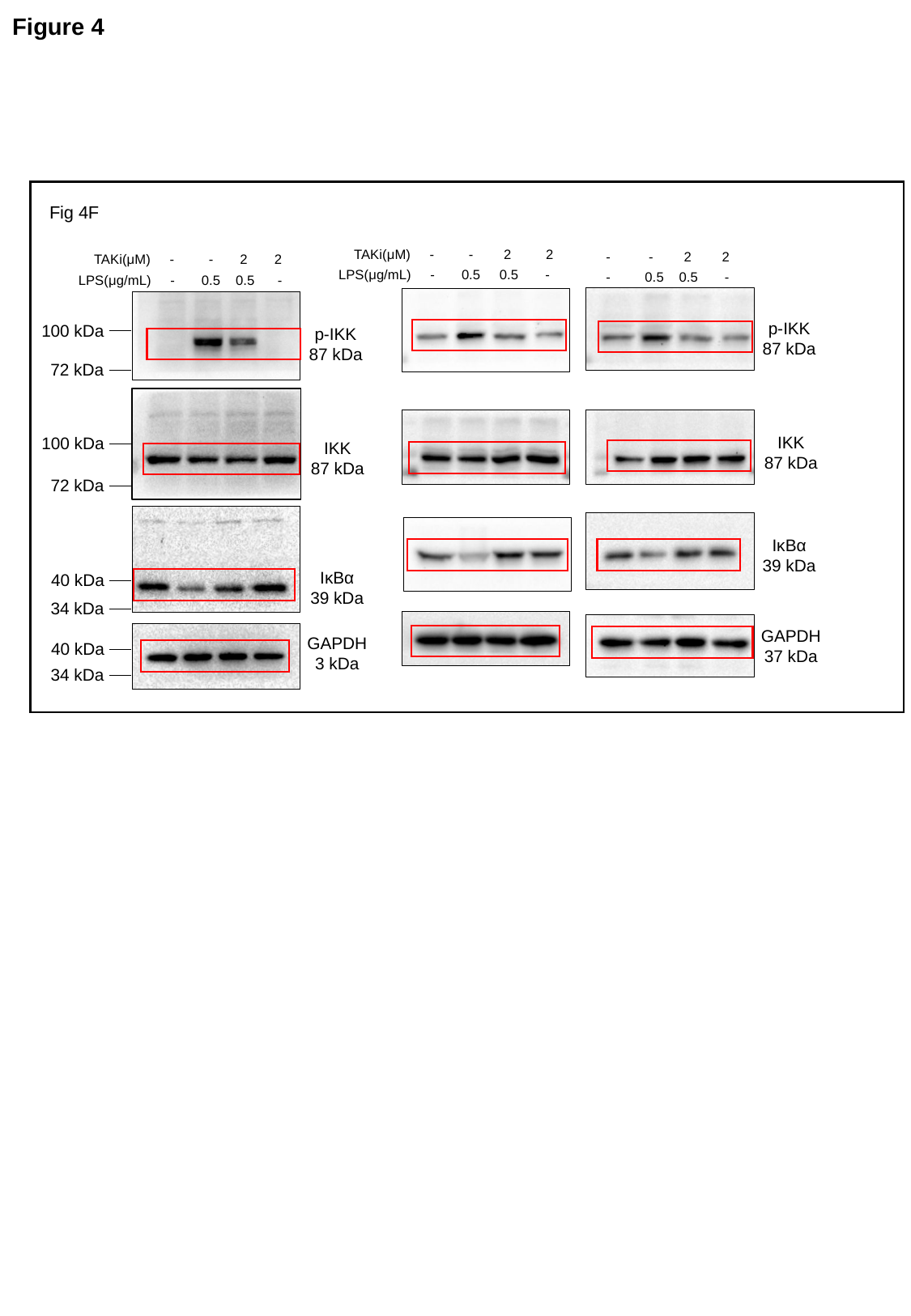

Figure 4
Fig 4F
 TAKi(μM) - - 2 2
- - 2 2
 TAKi(μM) - - 2 2
LPS(μg/mL) - 0.5 0.5 -
- 0.5 0.5 -
LPS(μg/mL) - 0.5 0.5 -
p-IKK
87 kDa
100 kDa
p-IKK
87 kDa
72 kDa
IKK
87 kDa
100 kDa
IKK
87 kDa
72 kDa
IκBα
39 kDa
IκBα
39 kDa
40 kDa
34 kDa
GAPDH
37 kDa
GAPDH
3 kDa
40 kDa
34 kDa

## Slide 14
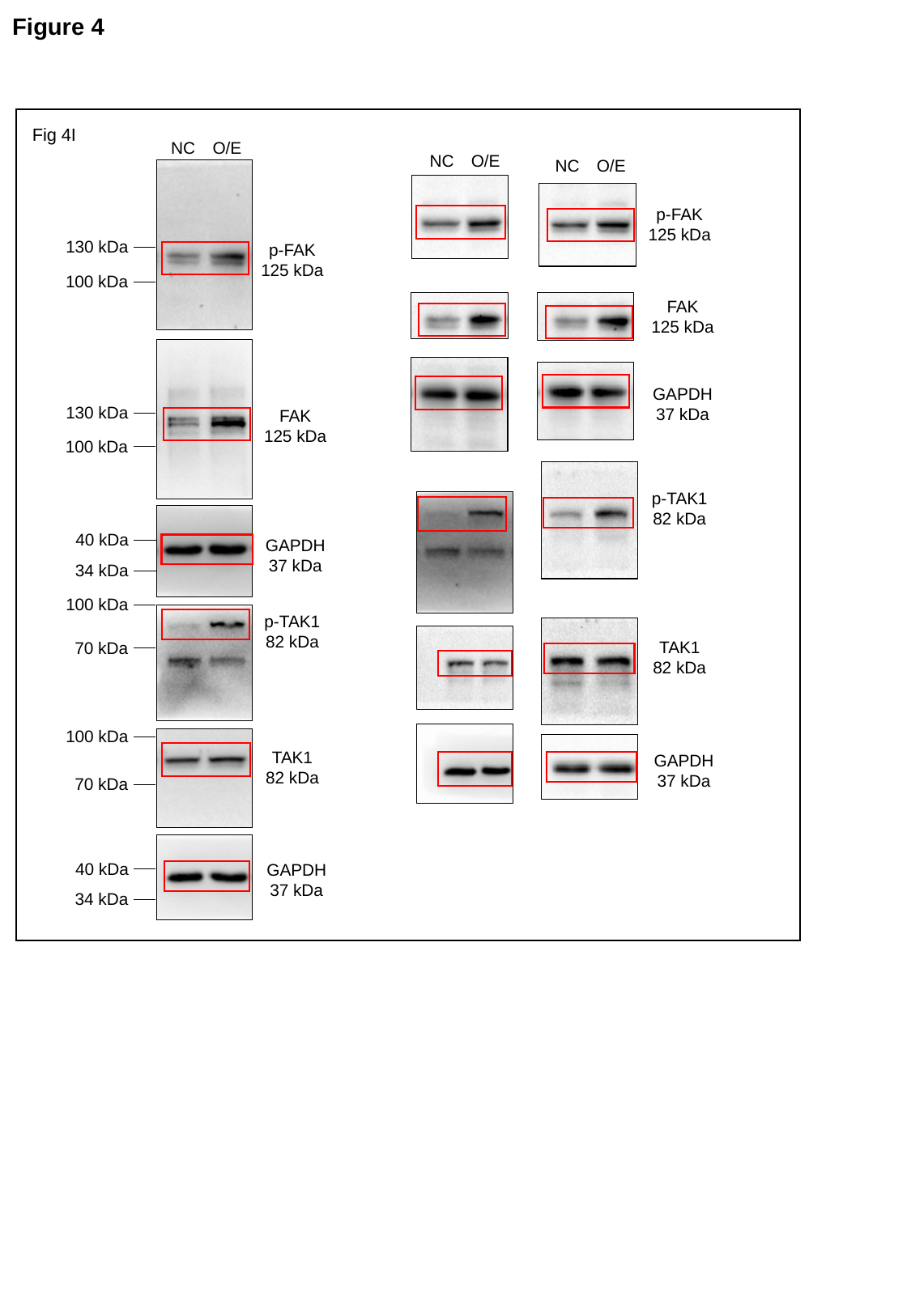

Figure 4
Fig 4I
O/E
NC
O/E
NC
O/E
NC
p-FAK
125 kDa
130 kDa
p-FAK
125 kDa
100 kDa
FAK
125 kDa
GAPDH
37 kDa
130 kDa
FAK
125 kDa
100 kDa
p-TAK1
82 kDa
40 kDa
GAPDH
37 kDa
34 kDa
100 kDa
p-TAK1
82 kDa
TAK1
82 kDa
70 kDa
100 kDa
TAK1
82 kDa
GAPDH
37 kDa
70 kDa
40 kDa
GAPDH
37 kDa
34 kDa

## Slide 15
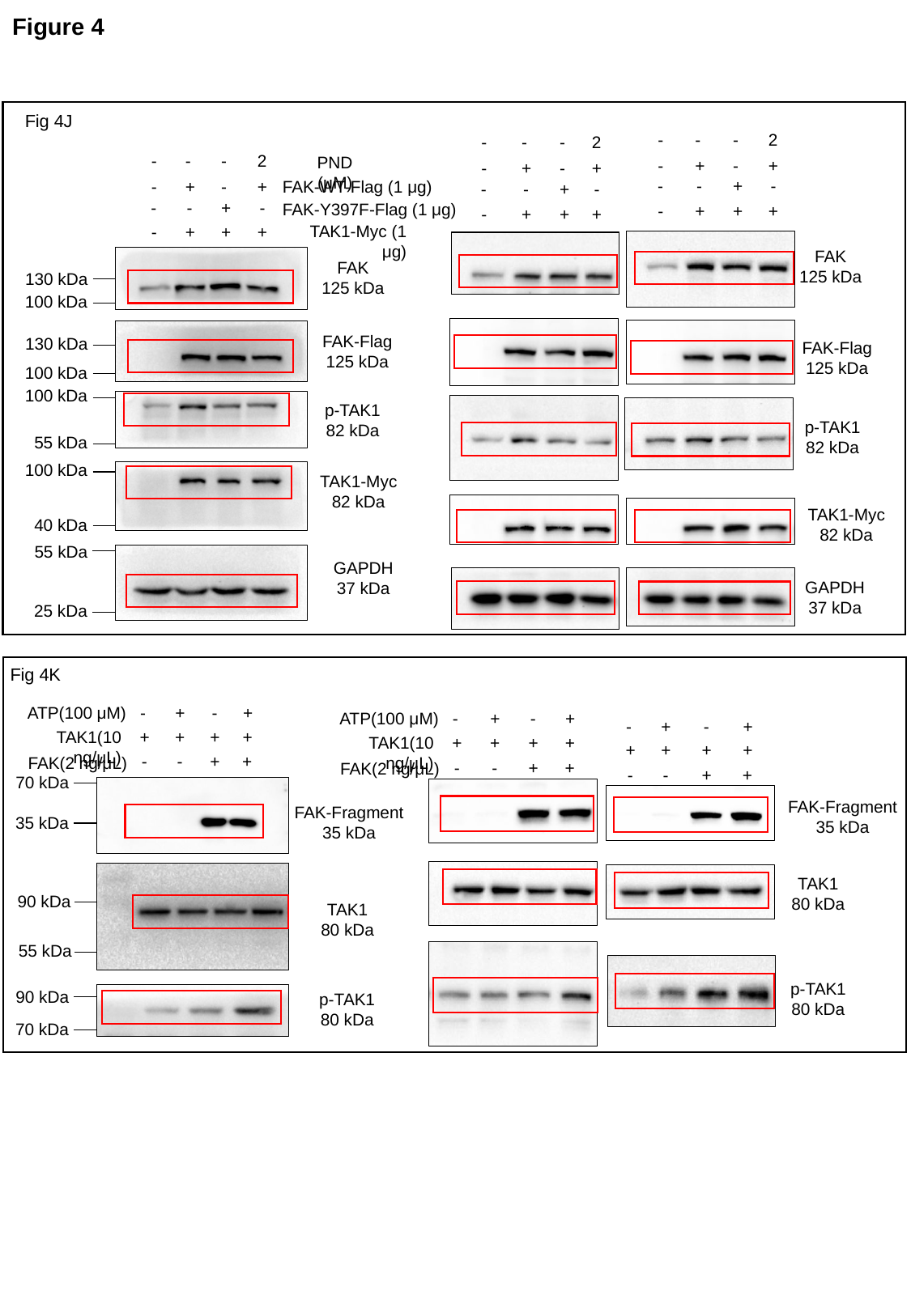

Figure 4
Fig 4J
-
-
-
2
-
-
-
2
-
-
-
2
PND (μM)
-
+
-
+
-
+
-
+
-
-
+
-
-
+
-
+
FAK-WT-Flag (1 μg)
-
-
+
-
-
-
+
-
FAK-Y397F-Flag (1 μg)
-
+
+
+
-
+
+
+
TAK1-Myc (1 μg)
-
+
+
+
FAK
125 kDa
FAK
125 kDa
130 kDa
100 kDa
FAK-Flag
125 kDa
130 kDa
FAK-Flag
125 kDa
100 kDa
100 kDa
p-TAK1
82 kDa
p-TAK1
82 kDa
55 kDa
100 kDa
TAK1-Myc
82 kDa
TAK1-Myc
82 kDa
40 kDa
55 kDa
GAPDH
37 kDa
GAPDH
37 kDa
25 kDa
Fig 4K
ATP(100 μM)
-
+
-
+
ATP(100 μM)
-
+
-
+
-
+
-
+
+
+
+
+
TAK1(10 ng/μL)
+
+
+
+
TAK1(10 ng/μL)
+
+
+
+
-
-
+
+
FAK(2 ng/μL)
-
-
+
+
FAK(2 ng/μL)
-
-
+
+
70 kDa
FAK-Fragment
35 kDa
FAK-Fragment
35 kDa
35 kDa
TAK1
80 kDa
90 kDa
TAK1
80 kDa
55 kDa
p-TAK1
80 kDa
90 kDa
p-TAK1
80 kDa
70 kDa

## Slide 16
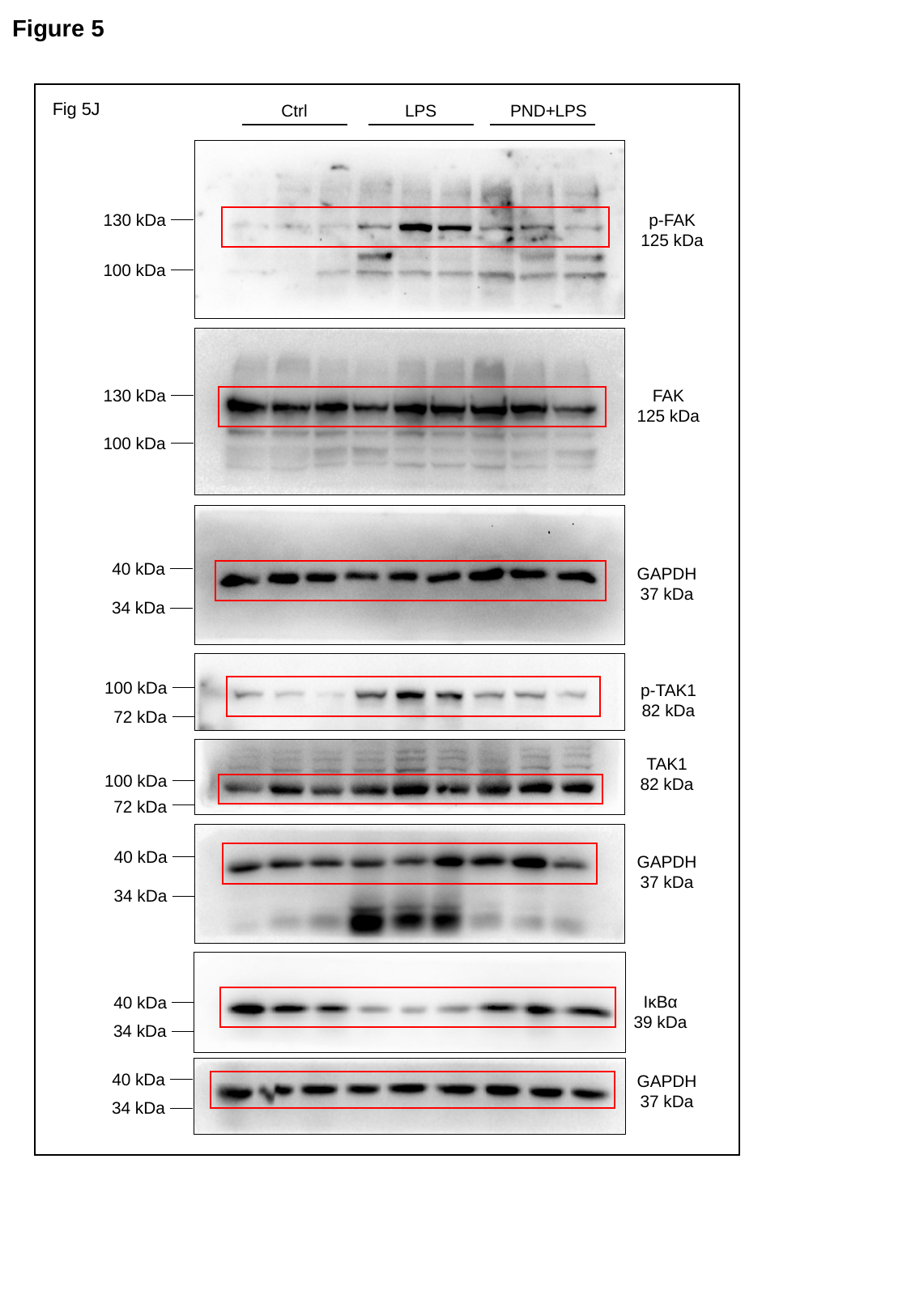

Figure 5
Fig 5J
Ctrl
LPS
PND+LPS
130 kDa
p-FAK
125 kDa
100 kDa
130 kDa
FAK
125 kDa
100 kDa
40 kDa
GAPDH
37 kDa
34 kDa
100 kDa
p-TAK1
82 kDa
72 kDa
TAK1
82 kDa
100 kDa
72 kDa
40 kDa
GAPDH
37 kDa
34 kDa
IκBα
39 kDa
40 kDa
34 kDa
40 kDa
GAPDH
37 kDa
34 kDa

## Slide 17
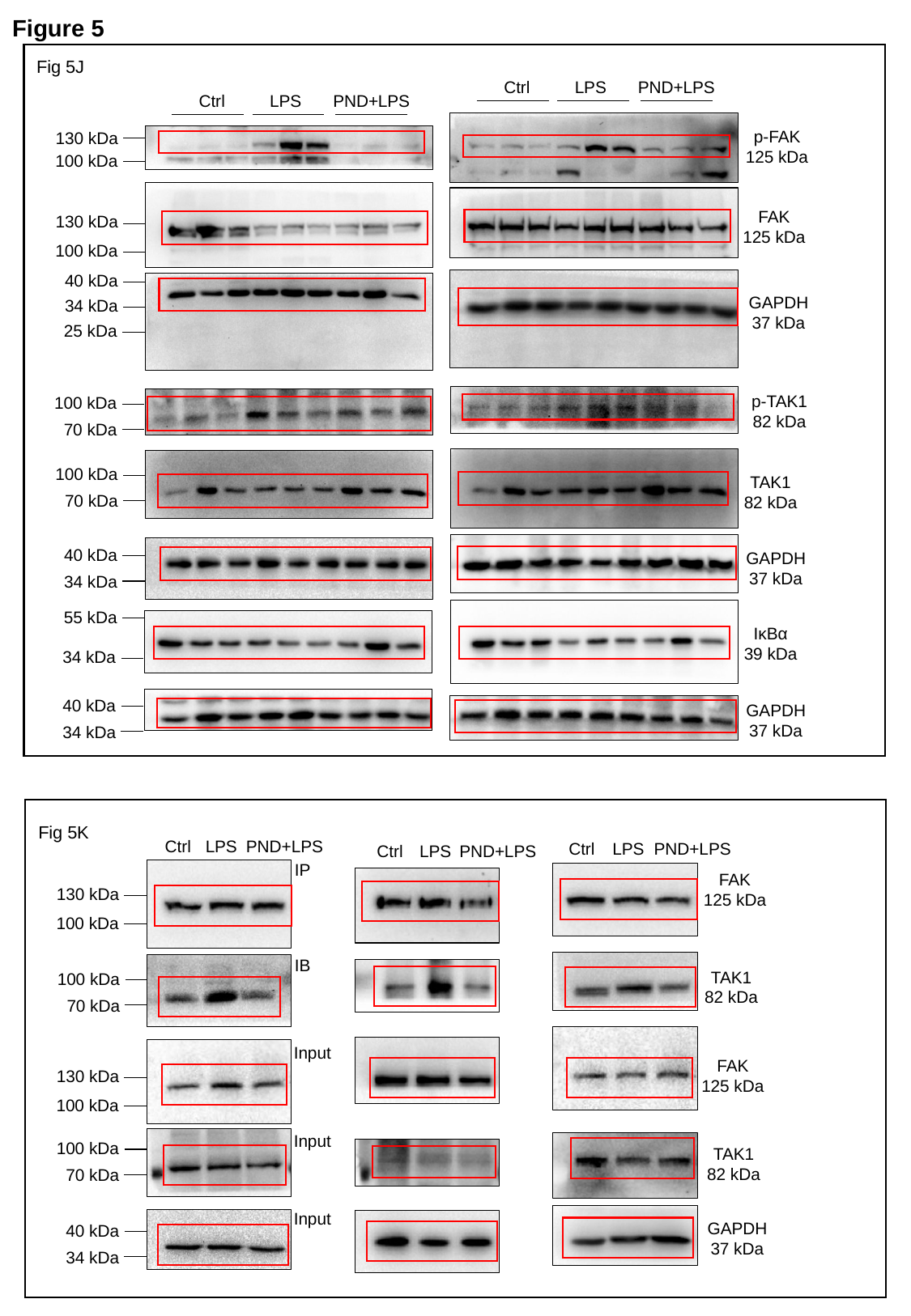

Figure 5
Fig 5J
Ctrl
LPS
PND+LPS
Ctrl
LPS
PND+LPS
p-FAK
125 kDa
130 kDa
100 kDa
FAK
125 kDa
130 kDa
100 kDa
40 kDa
GAPDH
37 kDa
34 kDa
25 kDa
p-TAK1
82 kDa
100 kDa
70 kDa
100 kDa
TAK1
82 kDa
70 kDa
40 kDa
GAPDH
37 kDa
34 kDa
55 kDa
IκBα
39 kDa
34 kDa
40 kDa
GAPDH
37 kDa
34 kDa
Fig 5K
Ctrl
LPS
PND+LPS
Ctrl
LPS
PND+LPS
Ctrl
LPS
PND+LPS
IP
FAK
125 kDa
130 kDa
100 kDa
IB
TAK1
82 kDa
100 kDa
70 kDa
Input
FAK
125 kDa
130 kDa
100 kDa
Input
100 kDa
TAK1
82 kDa
70 kDa
Input
GAPDH
37 kDa
40 kDa
34 kDa

## Slide 18
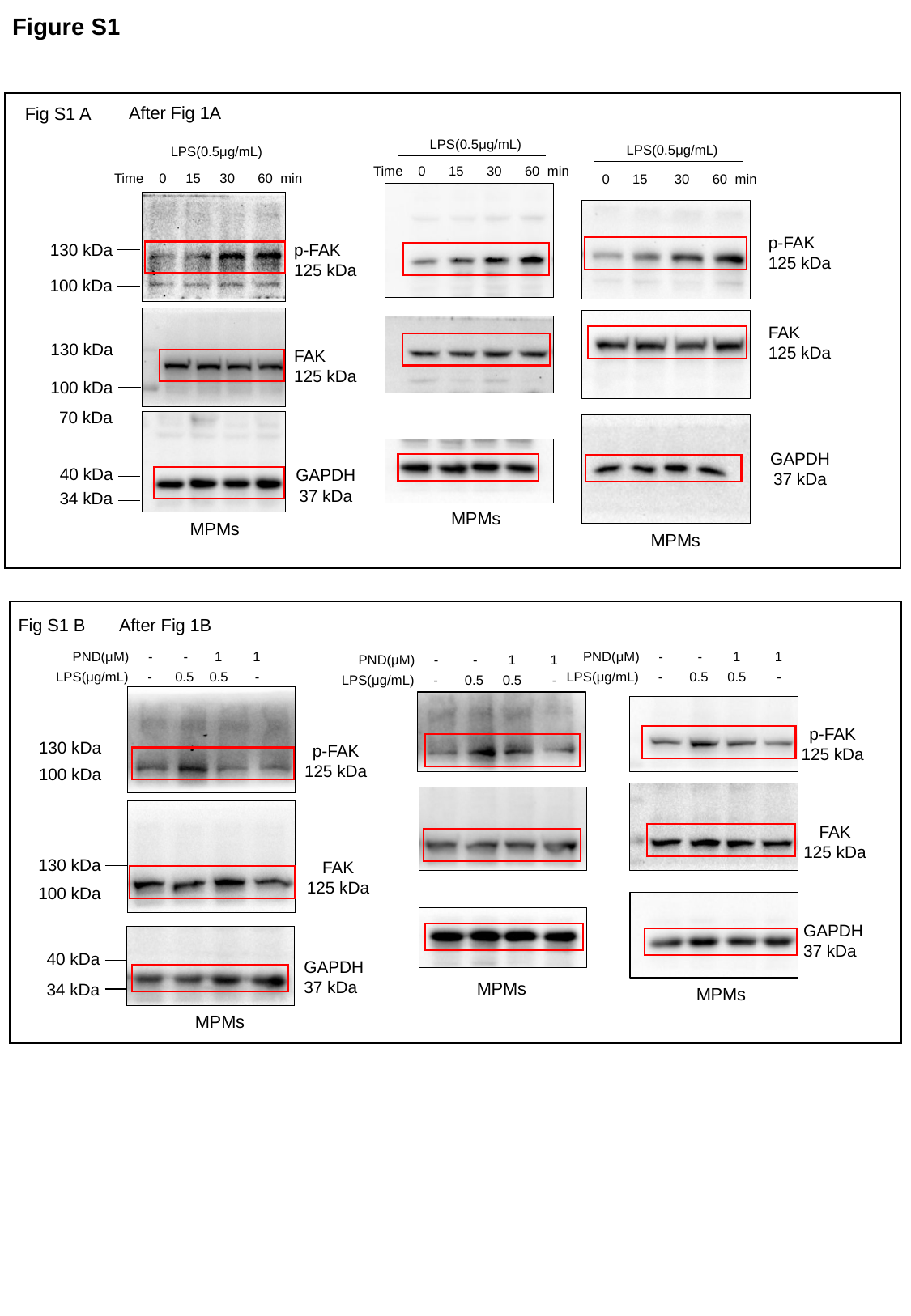

Figure S1
After Fig 1A
Fig S1 A
LPS(0.5μg/mL)
LPS(0.5μg/mL)
LPS(0.5μg/mL)
Time 0 15 30 60 min
Time 0 15 30 60 min
 0 15 30 60 min
p-FAK
125 kDa
130 kDa
p-FAK
125 kDa
100 kDa
FAK
125 kDa
130 kDa
FAK
125 kDa
100 kDa
70 kDa
GAPDH
37 kDa
40 kDa
GAPDH
37 kDa
34 kDa
MPMs
MPMs
MPMs
Fig S1 B
After Fig 1B
 PND(μM) - - 1 1
 PND(μM) - - 1 1
 PND(μM) - - 1 1
LPS(μg/mL) - 0.5 0.5 -
LPS(μg/mL) - 0.5 0.5 -
LPS(μg/mL) - 0.5 0.5 -
p-FAK
125 kDa
130 kDa
p-FAK
125 kDa
100 kDa
FAK
125 kDa
130 kDa
FAK
125 kDa
100 kDa
GAPDH
37 kDa
40 kDa
GAPDH
37 kDa
MPMs
34 kDa
MPMs
MPMs

## Slide 19
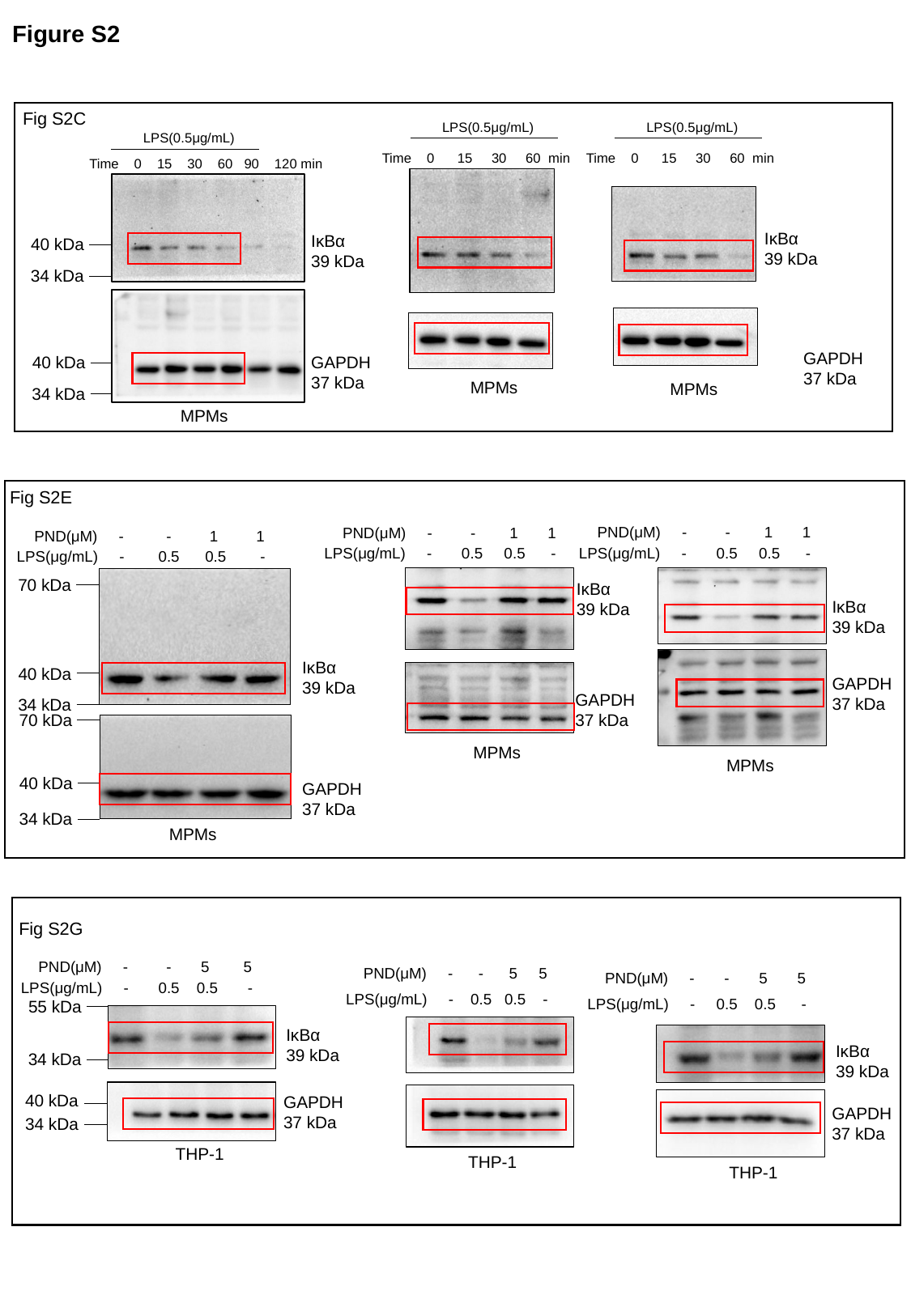

Figure S2
Fig S2C
LPS(0.5μg/mL)
LPS(0.5μg/mL)
LPS(0.5μg/mL)
Time 0 15 30 60 min
Time 0 15 30 60 min
Time 0 15 30 60 90 120 min
IκBα
39 kDa
IκBα
39 kDa
40 kDa
34 kDa
GAPDH
37 kDa
40 kDa
GAPDH
37 kDa
MPMs
MPMs
34 kDa
MPMs
Fig S2E
 PND(μM) - - 1 1
 PND(μM) - - 1 1
 PND(μM) - - 1 1
LPS(μg/mL) - 0.5 0.5 -
LPS(μg/mL) - 0.5 0.5 -
LPS(μg/mL) - 0.5 0.5 -
70 kDa
IκBα
39 kDa
IκBα
39 kDa
IκBα
39 kDa
40 kDa
GAPDH
37 kDa
GAPDH
37 kDa
34 kDa
70 kDa
MPMs
MPMs
40 kDa
GAPDH
37 kDa
34 kDa
MPMs
Fig S2G
 PND(μM) - - 5 5
 PND(μM) - - 5 5
 PND(μM) - - 5 5
LPS(μg/mL) - 0.5 0.5 -
LPS(μg/mL) - 0.5 0.5 -
LPS(μg/mL) - 0.5 0.5 -
55 kDa
IκBα
39 kDa
IκBα
39 kDa
34 kDa
40 kDa
GAPDH
37 kDa
GAPDH
37 kDa
34 kDa
THP-1
THP-1
THP-1

## Slide 20
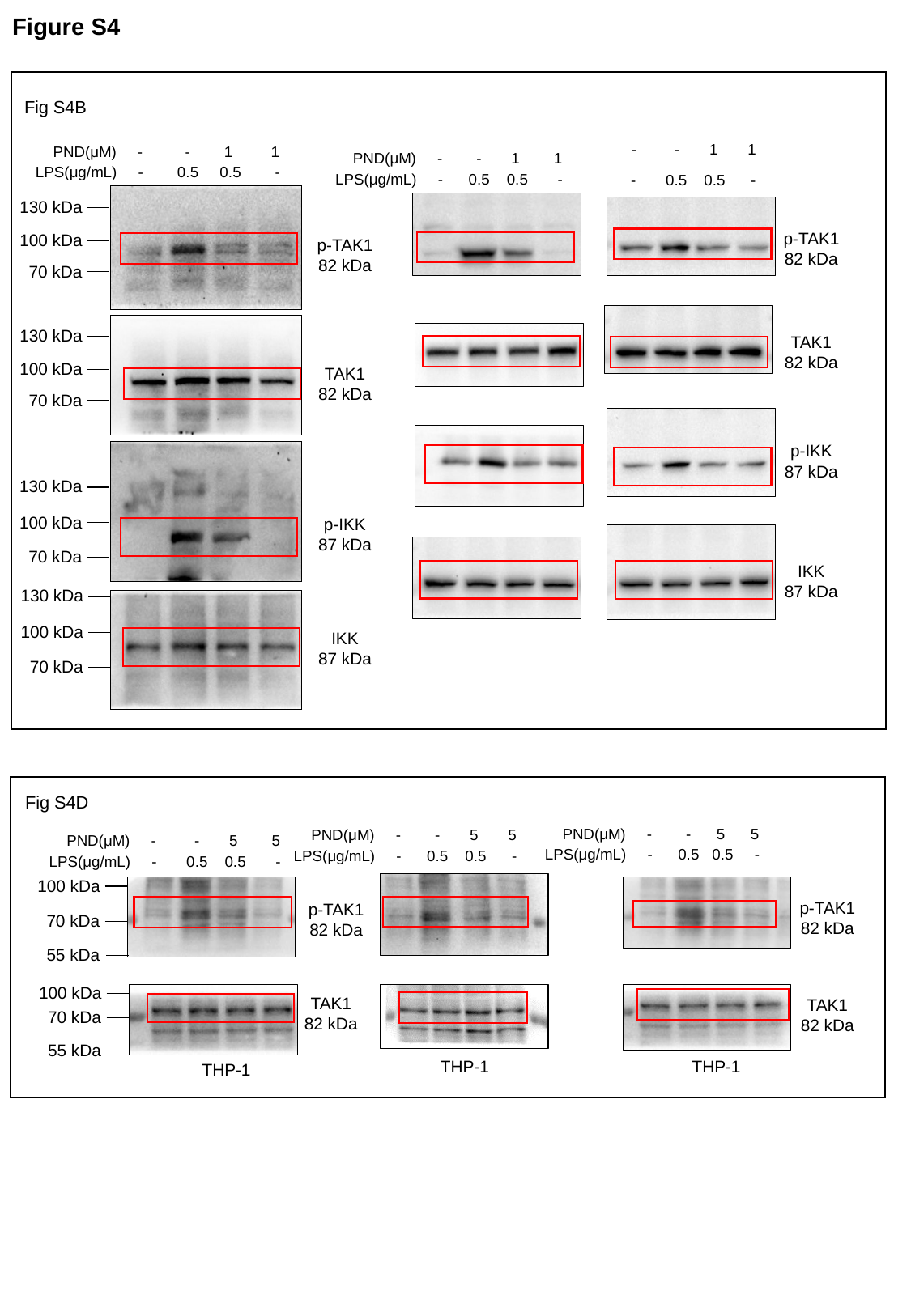

Figure S4
Fig S4B
- - 1 1
 PND(μM) - - 1 1
 PND(μM) - - 1 1
LPS(μg/mL) - 0.5 0.5 -
LPS(μg/mL) - 0.5 0.5 -
- 0.5 0.5 -
130 kDa
p-TAK1
82 kDa
100 kDa
p-TAK1
82 kDa
70 kDa
130 kDa
TAK1
82 kDa
100 kDa
TAK1
82 kDa
70 kDa
p-IKK
87 kDa
130 kDa
100 kDa
p-IKK
87 kDa
70 kDa
IKK
87 kDa
130 kDa
100 kDa
IKK
87 kDa
70 kDa
Fig S4D
 PND(μM) - - 5 5
 PND(μM) - - 5 5
 PND(μM) - - 5 5
LPS(μg/mL) - 0.5 0.5 -
LPS(μg/mL) - 0.5 0.5 -
LPS(μg/mL) - 0.5 0.5 -
100 kDa
p-TAK1
82 kDa
p-TAK1
82 kDa
70 kDa
55 kDa
100 kDa
TAK1
82 kDa
TAK1
82 kDa
70 kDa
55 kDa
THP-1
THP-1
THP-1
